# Deoxypyrimidine monophosphate bypass therapy for thymidine kinase 2 deficiency

**DOI:** 10.15252/emmm.201404092

**Published:** 2014-06-26

**Authors:** Caterina Garone, Beatriz Garcia-Diaz, Valentina Emmanuele, Luis C Lopez, Saba Tadesse, Hasan O Akman, Kurenai Tanji, Catarina M Quinzii, Michio Hirano

**Affiliations:** 1 Department of Neurology, Columbia University Medical CenterNew York, NY, USA; 2 Human Genetics Joint PhD Program, University of Bologna and TurinTurin, Italy; 3 Pediatric Clinic, University of Genoa, IRCCS G. Gaslini InstituteGenoa, Italy; 4 Instituto de Biotecnología, Centro de Investigación Biomédica, Universidad de Granada, Parque Tecnológico de Ciencias de la SaludArmilla, Spain; 5 Department of Pathology and Cell Biology, Columbia University Medical CenterNew York, NY, USA

**Keywords:** deoxycytidine monophosphate, deoxythymidine monophosphate, encephalomyopathy, therapy, thymidine kinase

## Abstract

Autosomal recessive mutations in the thymidine kinase 2 gene (*TK2*) cause mitochondrial DNA depletion, multiple deletions, or both due to loss of TK2 enzyme activity and ensuing unbalanced deoxynucleotide triphosphate (dNTP) pools. To bypass Tk2 deficiency, we administered deoxycytidine and deoxythymidine monophosphates (dCMP+dTMP) to the *Tk2* H126N (*Tk2*^*−/−*^) knock-in mouse model from postnatal day 4, when mutant mice are phenotypically normal, but biochemically affected. Assessment of 13-day-old *Tk2*^*−/−*^ mice treated with dCMP+dTMP 200 mg/kg/day each (*Tk2*^*−/−*200dCMP/^^dTMP^) demonstrated that in mutant animals, the compounds raise dTTP concentrations, increase levels of mtDNA, ameliorate defects of mitochondrial respiratory chain enzymes, and significantly prolong their lifespan (34 days with treatment versus 13 days untreated). A second trial of dCMP+dTMP each at 400 mg/kg/day showed even greater phenotypic and biochemical improvements. In conclusion, dCMP/dTMP supplementation is the first effective pharmacologic treatment for Tk2 deficiency.

**Subject Categories** Genetics, Gene Therapy & Genetic Disease; Metabolism

## Introduction

Encoded by the nuclear DNA gene *TK2*, thymidine kinase 2 (TK2) is a mitochondrial matrix protein that phosphorylates thymidine and deoxycytidine pyrimidine nucleosides to generate deoxythymidine monophosphate (dTMP) and deoxycytidine monophosphate (dCMP), which, in turn, are converted to deoxynucleotide triphosphates (dNTPs) required for mitochondrial DNA (mtDNA) synthesis. Autosomal recessive *TK2* mutations cause severe mtDNA depletion and devastating neuromuscular diseases in infants and children, as well as mtDNA multiple deletions and progressive external ophthalmoplegia in adults (Saada *et al*, 2001; Tyynismaa *et al*, 2012).

To elucidate the molecular pathogenesis of TK2 deficiency, we generated a homozygous *Tk2* H126N knock-in mutant (*Tk2*^*−/−*^) mouse that manifests a phenotype strikingly similar to the human infantile encephalomyopathy (Akman *et al*, 2008). Between postnatal day 10 and 13, *Tk2*^*−/−*^ mice rapidly develop fatal encephalomyopathy beginning with decreased ambulation, unstable gait, coarse tremor, and growth retardation that rapidly progress to early death at age 14–16 days (Dorado *et al*, 2011). A similar phenotype was observed in the *Tk2* knockout mouse (Zhou *et al*, 2008).

In the *Tk2*^*−/−*^ mice, loss of Tk2 activity caused dNTP pool imbalances with low dTTP levels in brain and decreased dTTP and dCTP in liver, which, in turn, produce mtDNA depletion and defects of mitochondrial respiratory chain (RC) complexes I, III, IV, and V containing mtDNA-encoded subunits, most prominently in the brain and spinal cord (Dorado *et al*, 2011).

Based on the understanding of the pathogenesis of Tk2 deficiency, we have assessed a rationale therapeutic strategy to bypass the enzymatic defect with oral dCMP and dTMP supplementation.

## Results

### dCMP/dTMP delays disease onset, prevents neuromuscular manifestations, and prolongs lifespan of Tk2-deficient mice

**Figure 1:**
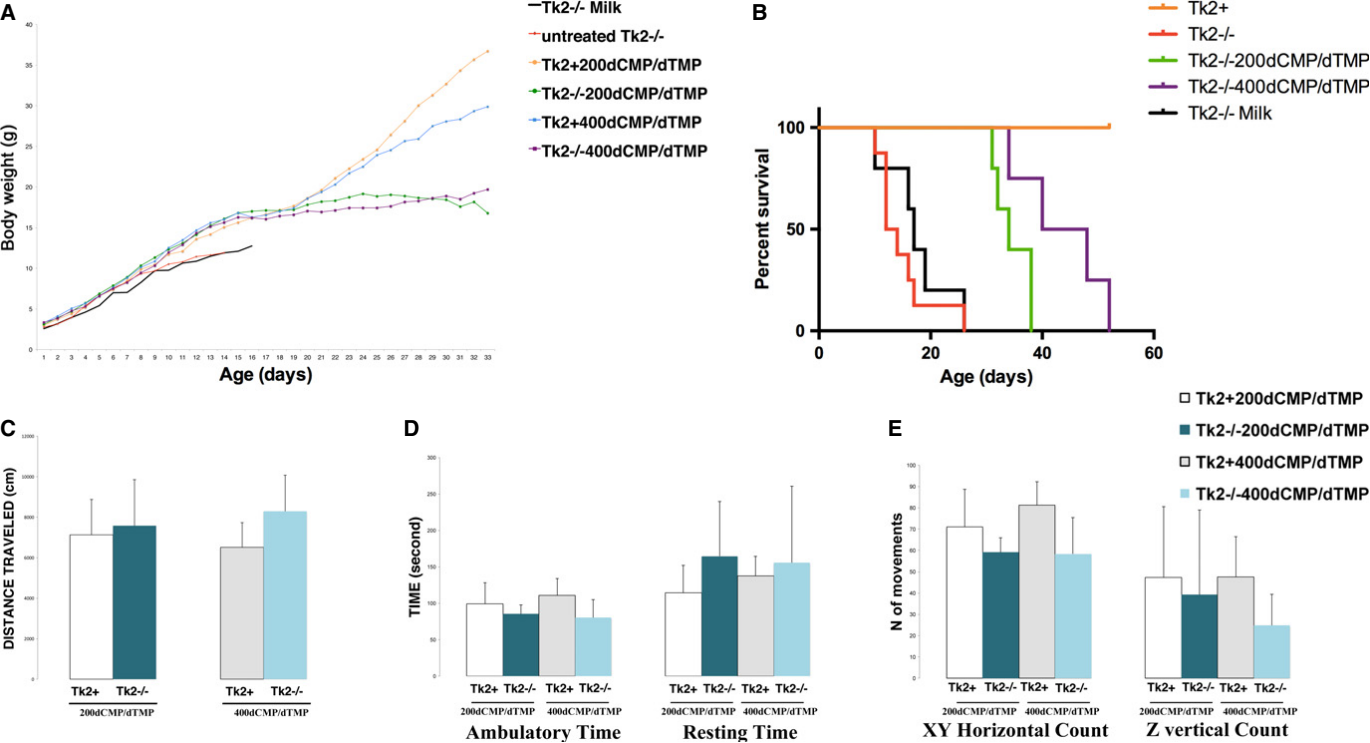
dCMP/dTMP efficacy on clinical phenotype A, B   Significant dose-related effects of the dCMP/dTMP on body weight (A) and survival (B) of mutant mice (*n* = 7 for each group)(*Tk2*^*−/−*^ versus *Tk2*^*−/−*200dCMP/^^dTMP^, *P* < 0.005; *Tk2*^*−/−*^ versus *Tk2*^*−/−*400dCMP/^^dTMP^, *P* < 0.005; Gehan-Breslow-Wilcoxon test). C–E   Open-field test in 29-day-old treated mice showing no difference in average distance traveled (C), ambulatory and resting times (D), and horizontal (XY axes) and vertical (Z-axis) movements (E) over 10 min in 29-day-old mutant and control mice treated with 200 mg/kg/day or 400 mg/kg/day of dCMP/dTMP (*n* = 5) (Data expressed as mean ± SD. Statistical analysis were performed on *Tk2*^−/−200dCMP/^^dTMP^ versus *Tk2*^*+*200dCMP/^^dTMP^ and *Tk2*^−/−400dCMP/^^dTMP^ versus *Tk2*^*+*400dCMP/^^dTMP^).

Oral treatment with dCMP+dTMP 200 mg/kg/day each in milk (*Tk2*^*−/−*200dCMP/dTMP^) beginning at postnatal day 4 delayed disease onset to 20–25 days (Supplementary Video S1), when the mutant mice developed a mild tremor and stopped gaining weight. In the fourth week, they manifested weakness and reduced movements. In contrast, *Tk2*^*−/−*^ mice treated from day 4 with dCMP+dTMP 400 mg/kg/day each in milk (*Tk2*^*−/−*400dCMP/dTMP^) appeared normal until day 21, when weight gain decelerated and mild head tremor developed (Fig 1A). Untreated *Tk2*^*−/−*^ mice had a mean lifespan of 13.2 ± 2.5 days (mean ± SD), whereas *Tk2*^*−/−*200dCMP/dTMP^ survived to 34.6 ± 3.2 days (*P* = 0.0028; *n* = 7; Gehan–Breslow–Wilcoxon test) while *Tk2*^*−/−*400dCMP/dTMP^ lived to 44.3 ± 9.1 days (*P* = 0.0071; *n* = 7; Gehan–Breslow–Wilcoxon test) (Fig 1B). The cause of death was not evident in postmortem histological studies of major organs in 29-day-old *Tk2*^*−/−*200dCMP/dTMP^ mice. No adverse side effects, including malignancies, were observed in the treated homozygous, and heterozygous wild types (*Tk2*^*+*^) and mutants except mild deceleration of weight gain in *Tk2*^*+*400dCMP/dTMP^ (Supplementary Table S1).

Open-field assessment of motor function in 29-day-old *Tk2*^*−/−*200dCMP/dTMP^, *Tk2*^*−/−*400dCMP/dTMP^, and wild-type *Tk2* mice showed no differences in the distance traveled, horizontal and vertical movements, or resting time (Fig 1C–E). Relative to 29-day-old *Tk2*^*+*^ mice, age-matched *Tk2*^*−/−*200dCMP/dTMP^ and *Tk2*^*−/−*400dCMP/dTMP^ animals showed decreases in gross muscle mass and muscle fiber diameter that were independent of the treatment dose but paralleled to body weight (Supplementary Fig S1). Histology showed no signs of myopathy or mitochondrial abnormalities (Supplementary Fig S1A–D). Biochemical studies demonstrated normal mitochondrial RC activities and protein levels (Supplementary Fig S1D–F).

### Histological and histochemical CNS studies confirmed dCMP/dTMP efficacy

**Figure 2:**
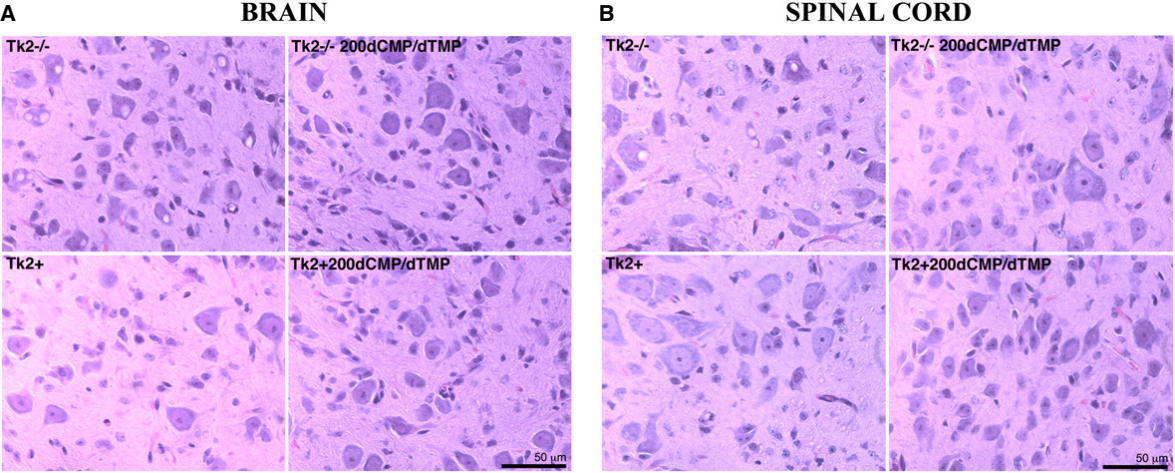
dCMP/dTMP effects on brain and spinal cord morphology A, B   Hematoxylin and eosin stain showing numerous vacuoles in 13-day-old untreated *Tk2*^*−/−*^ in brain (A) and spinal cord neurons (B). Vacuoles were rare or absent in *Tk2*^−/−200dCMP/^^dTMP^ and not observed in wild-type mice.

**Figure 3:**
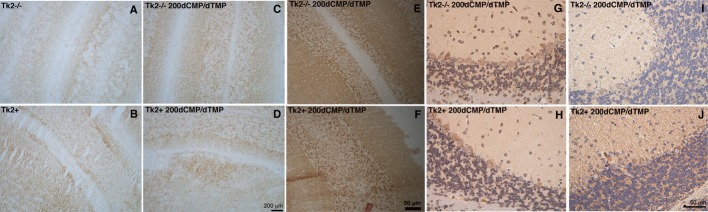
Complex I immunohistochemistry and complex IV histochemistry of cerebellum A–D   Complex IV (COX) histochemistry of cerebellum showing deficiency in 13-day-old untreated *Tk2*^*−/−*^ (A) in contrast to normal COX activity in *Tk2*^*+*^ (B), *Tk2*^−/−200dCMP/^^dTMP^ (C), and *Tk2*^*+*200dCMP/^^dTMP^ (D) mice. E–H   COX histochemistry (E-F) and immunostaining against COX subunit II (G-H) of cerebellum showed no differences between 29-day-old *Tk2*^−/−200dCMP/^^dTMP^ (upper panels) and age-matched *Tk2*^*+*200dCMP/^^dTMP^ mice (lower panels). I–J   Anti-complex I NDUFB8 subunit immunostaining of brain showed reduced staining in 29-day-old *Tk2*^−/−200dCMP/^^dTMP^ (I) versus *Tk2*^*+*200dCMP/^^dTMP^ mice (J).

Efficacy of treatment in central nervous system (CNS) was demonstrated in histological studies that showed dramatic reductions in the numbers of vacuoles in neurons of the spinal cord and cerebellar and brain stem nuclei of 13-day-old *Tk2*^*−/−*200dCMP/dTMP^ mice relative to untreated 13-day-old *Tk2*^*−/−*^mice (Fig 2A and B). Furthermore, cytochrome *c* oxidase (COX, complex IV) histochemistry of cerebellum revealed reduced overall COX activity in 13-day-old untreated *Tk2*^*−/−*^mice (Fig 3A) with normal activities in 13- and 29-day-old *Tk2*^*−/−*200dCMP/dTMP^ (Fig 3C and E) relative to *Tk2*^+^ animals (Fig 3B, D and F). No cell-specific immunohistochemical differences in COX protein were detected (Fig 3G and H) while severe reduction in complex I was identified by immunostaining of cerebellum of 29-day-old *Tk2*^*−/−*200dCMP/dTMP^ (Fig 3I and J).

### Treatment crosses biological barriers

To confirm that the treatment crosses biological barriers, we assessed dNTP levels in isolated mitochondria. In 13-day-old untreated *Tk2*^*−/−*^ mice relative to *Tk2*^*+*^ littermates, isolated brain mitochondria showed decreased levels of dTTP (0.67 ± 0.1 pmol/mg-protein versus 2.52 ± 1.0), while isolated liver mitochondria revealed reduced dCTP levels (1.07 ± 0.8 versus 2.9 ± 1.0) (Supplementary Table S2). The treatment crossed the blood–brain barrier increasing the level of dTTP in isolated brain mitochondria of 13-day-old *Tk2*^*+*200dCMP/dTMP^ (3.55 ± 1) and *Tk2*^*−/−*200dCMP/dTMP^ (1.5 ± 0.7) and as a consequence, restored the proportion of dTTP relative to total dNTP in treated mutants. In contrast, levels of dCTP in isolated mitochondria were stable in brain of 13-day-old *Tk2*^*+*200dCMP/dTMP^ (3.07 ± 2), decreased in brain of *Tk2*^*−/−*200dCMP/dTMP^ (1.13 ± 0.5), and decreased in liver of 13-day-old *Tk2*^*+*200dCMP/dTMP^ (1.13 ± 0.4) and *Tk2*^*−/−*200dCMP/dTMP^ (0.56 ± 0.5) (Supplementary Table S2).

**Figure 4:**
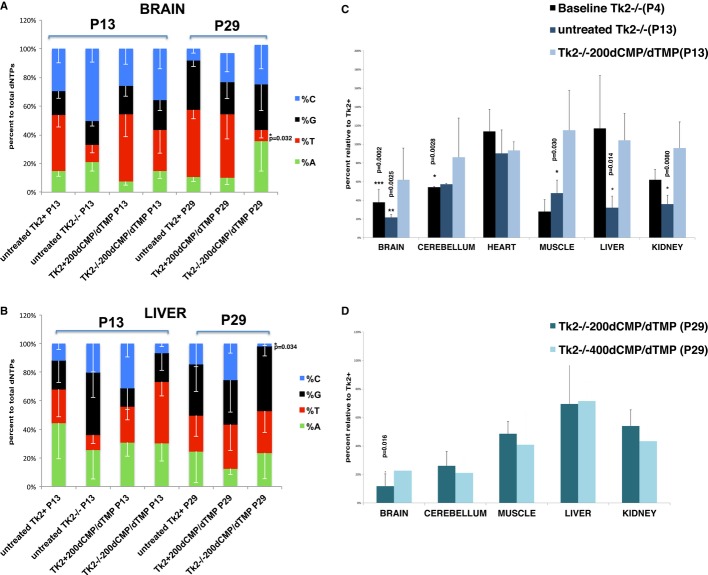
dCMP/dTMP effects on dNTP pool balance and mtDNA copy number A, B   Proportions of dNTPs (in percents) in isolated mitochondria of brain (A) and liver (B) of 13 and 29 postnatal day mice (P13 and P29) demonstrate that levels of dTTP (red sections) were increased in treated mutant versus untreated mutant mice at P13, but were severely decreased in P29 *Tk2*^−/−200dCMP/^^dTMP^ versus *Tk2*^*+*200dCMP/^^dTMP^ (*P < 0.05; ***P < 0.0005; Mann–Whitney *U*-test). C   mtDNA copy numbers in mice reveal rescue of mtDNA depletion in all tissues of treated mutants at P13 (*Tk2*^−/−^ versus *Tk2*^−/−200dCMP/^^dTMP^; *P < 0.05; **P < 0.005; ***P < 0.0005; Mann–Whitney *U*-test). D   Dose-related increase in mtDNA copy number in cerebral hemispheres of mutant mice at P29 (expressed as percent of untreated *Tk2*^*+*^ controls; mean ± SD; *Tk2*^−/−200dCMP/^^dTMP^ versus *Tk2*^−/−400dCMP/^^dTMP^; *P < 0.05; Mann–Whitney *U*-test) (*n* = 5 for each group). Source data is available online for this figure.

In 29-day-old *Tk2*^*−/−*200dCMP/dTMP^ relative to *Tk2*^*+*^ mice, absolute levels of dTTP and dCTP were markedly reduced in isolated mitochondria from brain (dTTP 0.11 ± 0.05 and dCTP 0.6 ± 0.2) and from liver (dTTP 0.15 ± 0.04 and dCTP 0.04 ± 0.03) (Supplementary Table S2); when these data were expressed as percentage of total dNTPs, there were striking decreases in dTTP/dNTP in brain (*P* = 0.0322; *n* = 7; Mann–Whitney *U*-test) and dCTP/dNTP in liver (*P* = 0.0338; *n* = 3; Mann–Whitney *U*-test) (Fig 4A and B).

### dCMP/dTMP treatment ameliorates biochemical and molecular genetic abnormalities

Treatment with dCMP and dTMP enhanced mtDNA levels in the mutant mice. At pre-treatment baseline, 4-day-old *Tk2*^*−/−*^ mice did not manifest clinical abnormalities, but showed reductions of mtDNA copy numbers in brain cerebrum (38 ± 13% mtDNA relative to wild-type brain, *P* = 0.0002; *n* = 5; Mann–Whitney *U*-test), cerebellum (54 ± 1%, *P* = 0.0228; *n* = 4; Mann–Whitney *U*-test), muscle (28 ± 12%), and kidney (62 ± 11%) with normal mtDNA levels in heart and liver (Fig 4C). At age 13 days, untreated *Tk2*^*−/−*^ animals showed marked mtDNA depletion in brain cerebrum (21 ± 3%, *P* < 0.0025; *n* = 5; Mann–Whitney *U*-test), muscle (47 ± 1%, *P* = 0.0303; *n* = 7; Mann–Whitney *U*-test), liver (32 ± 1%, *P* = 0.0140; *n* = 5; Mann–Whitney *U*-test), and kidney (35 ± 9%, *P* = 0.008; *n* = 6; Mann–Whitney *U*-test), but stable mtDNA depletion in the cerebellum (Fig 4C). In contrast, with treatment, 13-day-old *Tk2*^*−/−*200dCMP/dTMP^ mice manifested moderate mtDNA depletion only in brain cerebrum (66 ± 34%) and normal mtDNA levels in cerebellum, muscle, heart, liver, and kidney (Fig 4C).

At age 29 days, relative to *Tk2*^*+*^, *Tk2*^*−/−*200dCMP/dTMP^ mice showed mtDNA depletion that was severe in cerebellum (23 ± 8%) and brain cerebrum (11 ± 1%) and moderate in muscle (48 ± 23%), liver (70 ± 13%), and kidney (55 ± 6%) (Fig 4D). Compared with *Tk2*^*−/−*200dCMP/dTMP^ mice, *Tk2*^*−/−*400dCMP/dTMP^ animals had less severe mtDNA depletion in brain cerebrum (22 ± 8%, *P* = 0.0159; *n* = 6; Mann–Whitney *U*-test), but similar mtDNA depletion in muscle (40 ± 8%), liver (71 ± 36%), and kidney (43 ± 11%) and cerebellum (26 ± 12%) (Fig 4D).

**Figure 5:**
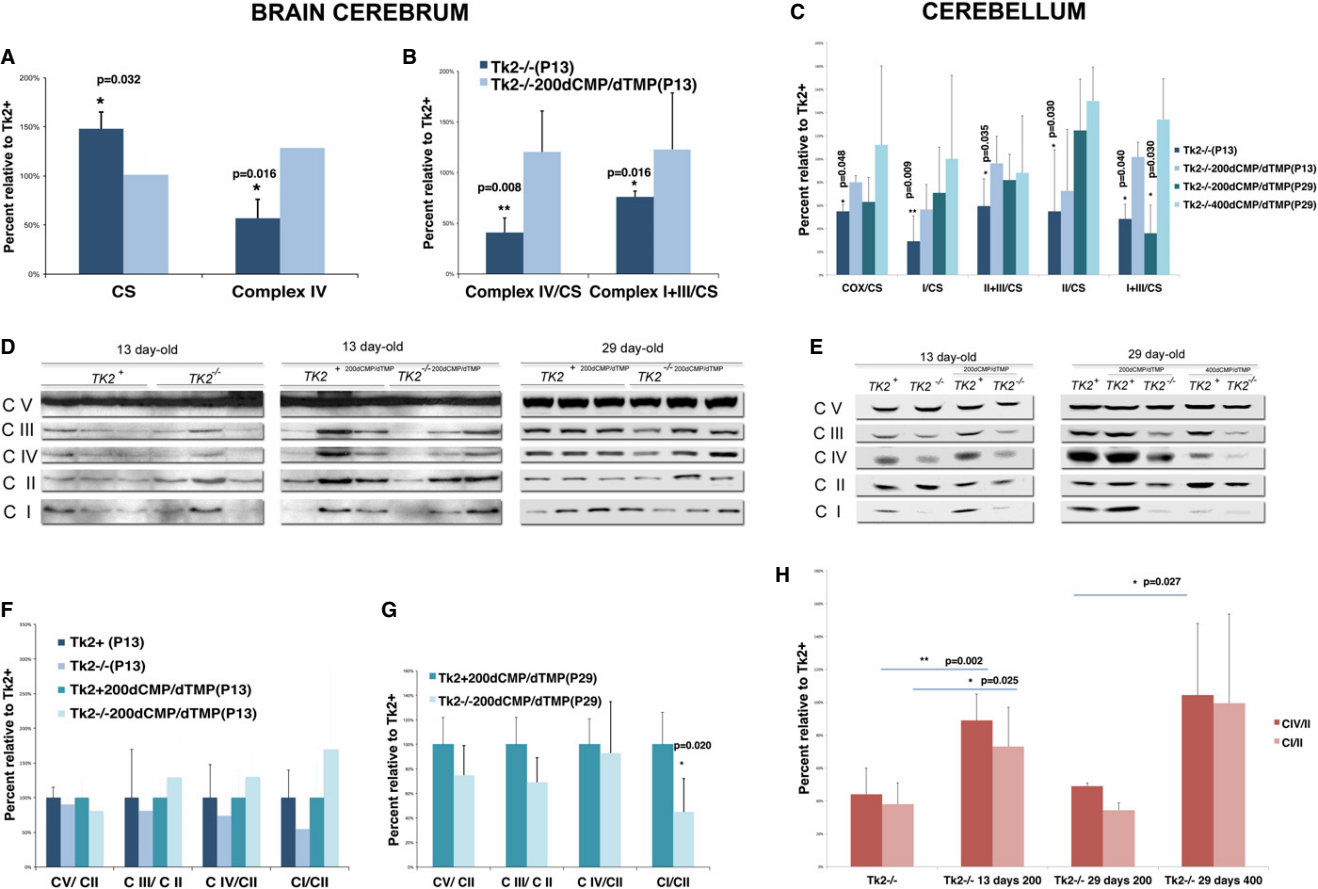
dCMP/dTMP efficacy on brain hemisphere and cerebellum biochemistry A, B   (A) Cerebral hemispheres of untreated *Tk2*^−/−^ mice relative to untreated wild types showed significant increase in CS activity and deficiency of complex IV activity (micromole/min/mg tissue normalized to mg-protein; mean ± SD) as well as (B) defects of complexes IV and I+III activities when referred to CS (mean ± SD). In contrast, with 200 mg/kg/day dCMP/dTMP, activities of citrate synthase and complex IV were normal in cerebrum of 13-day-old *Tk2*^−/−^. C   In cerebellum of mutant mice at ages 13 and 29 days, activities of mitochondrial RC referred to CS (expressed as percent of *Tk2*^*+*^) showed treatment-dose-related increases. D–E   Western blot of OXPHOS protein (MitoProfile® Total OXPHOS Rodent WB Antibody Cocktail, MitoSciences®) in brain (D) and cerebellum (E) of 13-day-old untreated *Tk2*^−/−^*,* 13- and 29-day-old *Tk2*^−/−200dCMP/^^dTMP^ mice, and cerebellum of *Tk2*^−/−400dCMP/^^dTMP^ (expressed as percents relative to *Tk2*^*+*^). F–H   Quantitation of western blot bands demonstrated that treatment normalized levels of complexes I and IV protein in both brain cerebrum and cerebellum tissues at 13 days, but did not correct complexes I and III deficiencies in brain cerebrum and complexes I and IV in cerebellum at 29 days of age. Statistical analyses were performed using *Tk2*^−/−^ versus *Tk2*^−/−200dCMP/^^dTMP^ with P13 cerebral samples (F); *Tk2*^−/−200dCMP/^^dTMP^ versus *Tk2*^*+*200dCMP/^^dTMP^ with P29 cerebral samples (G); *Tk2*^−/−^ versus *Tk2*^−/−200dCMP/^^dTMP^ with P13 cerebellar samples (H); and *Tk2*^−/−200dCMP/^^dTMP^ versus *Tk2*^−/−400dCMP/^^dTMP^ with P29 cerebellar samples (H) *P < 0.05; **P < 0.005. Data information: Statistical analyses were performed with Mann–Whitney *U*-test and Unpaired *t*-test with Welch's correction. CS, citrate synthase; IV, cytochrome *c* oxidase (COX); I+III, NADH-cytochrome *c* reductase; I, NADH-dehydrogenase; II, succinate dehydrogenase; III, cytochrome *c* reductase; V, ATP synthase; *P*, postnatal day.

To assess the impact of treatment on mitochondrial RC enzymes, their activities and steady-state protein levels in brain cerebrum and cerebellum were measured. In 13-day-old untreated *Tk2*^*−/−*^ mice, relative to untreated wild-type, brain cerebrum showed reduced COX activity (57 ± 19%, *P* = 0.0159; *n* = 5; Mann–Whitney *U*-test) and significantly increased citrate synthase (CS) activity (148 ± 17%; *P* = 0.0317; *n* = 5; Mann–Whitney *U*-test) (Fig 5A, Supplementary Table S3) and, when normalized to CS, revealed decreased activities of complexes I+III (NADH-cytochrome *c* reductase) (76 ± 0.06%, *P* = 0.0159; *n* = 5; Mann–Whitney *U*-test) and II+III (succinate-cytochrome *c* reductase) (72 ± 9%) in addition to IV (41 ± 14%, *P* = 0.0079; *n* = 5; Mann–Whitney *U*-test) (Fig 5B, Supplementary Table S3). The RC defects were more severe in cerebellum with significant reductions in all of the complexes when normalized either to CS (Fig 5C) or to mg-proteins with predominant defect in complex I (29 ± 15%; *P* = 0.0087; *n* = 5; Unpaired *t*-test with Welch's correction) and increased CS activity (129 ± 34%) (Supplementary Table S4). In contrast*,* 13-day-old *Tk2*^*−/−*200dCMP/dTMP^ had normal RC enzyme activities in brain cerebrum (Fig 5A and B, Supplementary Table S3) and only a mild defect in complex I (56 ± 21%) in cerebellum compared with age-matched treated control mice (Fig 5C, Supplementary Table S4). In 29-day-old *Tk2*^*−/−*200dCMP/dTMP^, activities of RC enzymes were normal in brain cerebrum (Supplementary Table S3). In contrast, cerebellum of *Tk2*^*−/−*200dCMP/dTMP^ manifested a mild defect in complex IV (62 ± 20%) and severe defect in complex I+III (35 ± 24%, *P* = 0.0296; *n* = 5; Mann–Whitney *U*-test), while RC activities were completely rescued in the *Tk2*^*−/−*400dCMP/dTMP^ (Fig 5C, Supplementary Table S4). Western blot analysis of mitochondrial RC complex subunits in 13-day-old untreated *Tk2*^*−/−*^ animals compared with untreated control mice revealed reductions in steady-state levels of complex I (55 ± 39% brain cerebrum; 38 ± 13% cerebellum, *P* = 0.025; *n* = 6; Mann–Whitney *U*-test) and complex IV (74 ± 32% brain cerebrum; 44 ± 16% cerebellum, *P* = 0.0017; *n* = 6; Mann–Whitney *U*-test), while RC protein levels were normal in 13-day-old *Tk2*^*−/−*200dCMP/dTMP^ mice (Fig 5D–F and H). In 29-day-old *Tk2*^*−/−*200dCMP/dTMP^ compared with *Tk2*^*+2*00dCMP/dTMP^, we observed reduced levels of subunits of complexes I (45% ± 2, *P* = 0.0196; *n* = 4; Mann–Whitney *U*-test) and III (69% ± 2) in brain cerebrum (Fig 5G) and complexes I (34% ± 4) and IV (49% ± 1, *P* = 0.0267; *n* = 4; Unpaired *t*-test with Welch correction) in cerebellum (Fig 5H). In 29-day-old *Tk2*^*−/−*400dCMP/dTMP^, we observed complete rescue of the RC defect when compared to *Tk2*^*+4*00dCMP/dTMP^.

### Deoxynucleotide metabolism

**Figure 6:**
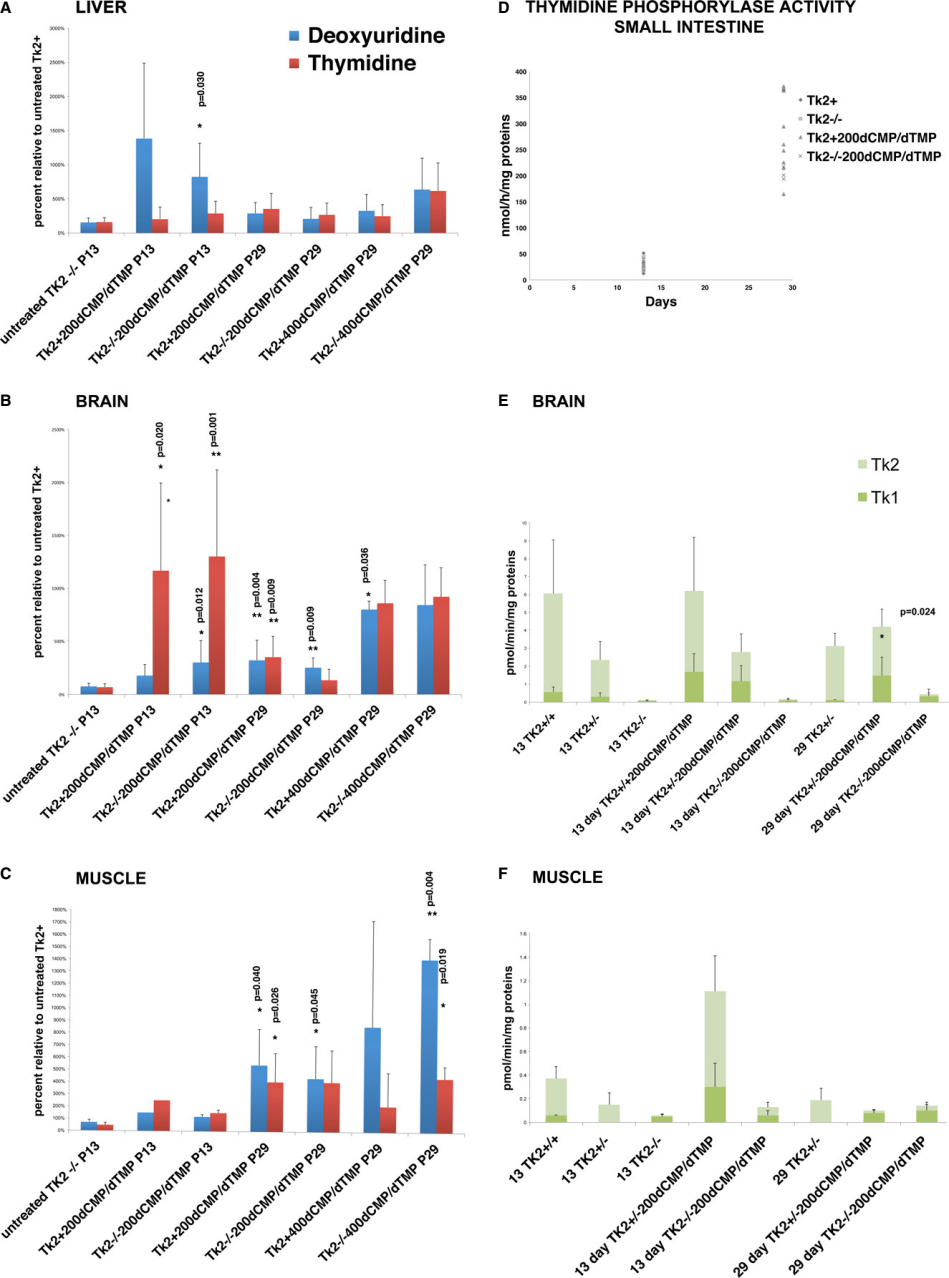
Metabolism of dCMP/dTMP A–C   Level of deoxyuridine and deoxythymidine in liver, brain, and muscle tissues was markedly higher at 13 days, but lower at 29 days. Deoxynucleoside levels are expressed as percents relative to age-matched untreated wild-type controls (mean ± SD) (*Tk2*^−/−200dCMP/^^dTMP^, *Tk2*^*+*200dCMP/^^dTMP^, *Tk2*^−/−400dCMP/^^dTMP^, and *Tk2*^*+*400dCMP/^^dTMP^ versus untreated *Tk2*^+^; *P < 0.05;**P < 0.005; Unpaired *t*-test with Welch's correction; *n* > 3 mice for each group). D   Thymidine phosphorylase (TP) activity in small intestine of treated and untreated *Tk2* mice showing dramatically increased activity at age 29 days relative to 13 days. Data expressed as nmol/h/mg-proteins (mean ± SD). E, F   Tk1 and Tk2 activities in brain and muscle tissues of treated and untreated mice showing increased Tk1 activity in treated mice. Data expressed in pmol/min/mg-proteins (mean ± SD) (*Tk2*^+/+^ versus *Tk2*^*+/+*200dCMP/^^dTMP^; *Tk2*^+/−^ versus *Tk2*^+/−200dCMP/^^dTMP^; *Tk2*^−/−^ versus *Tk2*^−/−200dCMP/^^dTMP^; *P < 0.05; Unpaired *t*-test with Welch's correction; *n* > 3 mice for each group). *P* = *p*-value.

To understand the metabolism of dCMP/dTMP after oral gavage administration, we measured levels of dCMP/dTMP and their metabolites in muscle and liver tissues and in plasma after 30 min of oral gavage. In *Tk2*^*−/−*200dCMP/dTMP^ mice, deoxynucleoside monophosphates were not detectable. Levels of deoxyuridine and deoxythymidine were markedly increased at age 13 days, but subsequently lower in 29-day-old mice (Fig 6A–C). Thymidine phosphorylase (TP) degrades deoxyuridine and deoxythymidine, respectively, to uracil and thymine plus deoxyribose 1-phosphate (Brown & Bicknell, 1998; Hirano *et al*, 2004). To understand the cause for differences in deoxyuridine and deoxythymidine plasma levels between 13 and 29 days of age, we measured the activity level of TP in small intestine, brain, and liver. TP activity was higher in the small intestine at P29 (Fig 6D), but unchanged in brain and liver tissues (Supplementary Table S5). Therefore, intestinal TP is responsible for the rapid catabolism of deoxyuridine and deoxythymidine at P29 and the resulting reduced plasma levels.

Tk2 activity was confirmed to be normal in treated and untreated *Tk2*^*+*^ mice and reduced in *Tk2*^*−/−*^ mice in muscle and brain. Unexpectedly, Tk1 activity was increased in brain and muscle of 13- and 29-day-old treated mice (Fig 6E and F).

## Discussion

Mitochondrial DNA depletion syndrome (MDS) is a frequent cause of severe childhood encephalomyopathy characterized molecularly by reduction of mtDNA copy number in tissues and insufficient synthesis of mitochondrial RC complexes (Hirano *et al*, 2001; Spinazzola & Zeviani, 2009). Mutations in eight nuclear genes have been identified as causes of infantile MDS (*TK2, DGUOK, POLG, MPV17, RRM2B, SUCLA2, SUCLG1,* and *C10orf2)* (Mandel *et al*, 2001; Saada *et al*, 2003; Naviaux & Nguyen, 2004; Elpeleg *et al*, 2005; Spinazzola *et al*, 2006; Bourdon *et al*, 2007; Ostergaard *et al*, 2007; Sarzi *et al*, 2007); 7 of the genes encode proteins involved in mtDNA replication or in the metabolism of deoxynucleotide triphosphate pools utilized as precursors for DNA replication (Copeland, 2012).

Treatment for MDS, like most mitochondrial disorders, has been limited to supportive therapies; however, understanding the pathomechanism of MDS enables the design of treatment strategies targeting either the cause of the disease or the downstream metabolic defects. Enzyme replacement by allogenic hematopoietic stem cell transplantation (HSCT) has shown promising initial results with MNGIE due to mutations in the *TYMP* gene encoding thymidine phosphorylase, another enzyme involved in nucleoside metabolism (Nishino *et al*, 1999; Hirano *et al*, 2006; Garone *et al*, 2011) but limited to disorders with toxic metabolites that can be eliminated by circulating cells. In contrast to AHSCT, gene therapy holds greater promise for mitochondrial diseases and other genetic disorders, but faces several barriers such as inefficient gene delivery, immune responses, and short-lived effects.

An alternative metabolic bypass approach has been tested in mtDNA-depleted myotubes from patients with *DGUOK* mutations. Remarkably, supplementation of culture media with deoxypurine nucleoside monophosphates (dAMP and dGMP), the products of dGK activity, partially restored mtDNA levels (Bulst *et al*, 2009) indicating that extracellular dAMP and dGMP are able to cross plasma and mitochondrial membranes and reach the mitochondrial matrix where they enter the nucleotide salvage pathway after bypassing the dGK defect. Concentrations of dAMP/dGMP up to 200–400 μM increased mtDNA levels in a dose-related fashion, while higher levels up to 1,200 μM (corresponding to 150 mg/kg/day in mice) did not further increase mtDNA or cause nuclear DNA or mtDNA qualitative defects such as chromosomal rearrangement or mtDNA deletions (Bulst *et al*, 2009).

We tested dCMP and dTMP supplementation in our *Tk2* knock-in mouse model to bypass the Tk2 defect. Deoxypyrimidine nucleoside monophosphate supplementation delayed the disease onset, reduced the severity of phenotypic manifestations, and prolonged the survival of the mutant mice in a dose-related manner. No adverse side effects, including malignancies, were observed. Oral dTMP/dCMP crossed biological barriers including the blood–brain barrier (BBB) because treatment increased dTTP in brain and liver in 13-day-old *Tk2*^*−/−*^ mice and augmented levels of mtDNA restoring the mitochondrial RC activities and protein defects in brain, heart, muscle, liver, and kidney of 13- and 29-day-old mutant mice. Treatment-related marked improvements of mtDNA levels and biochemical defects in muscle, which is the most affected tissue in *TK2* mutant patients, suggest that dCMP/dTMP might be more efficacious in patients with myopathy due to TK2 deficiency than in *Tk2*^*−/−*^ mice with severe CNS involvement.

**Figure 7:**
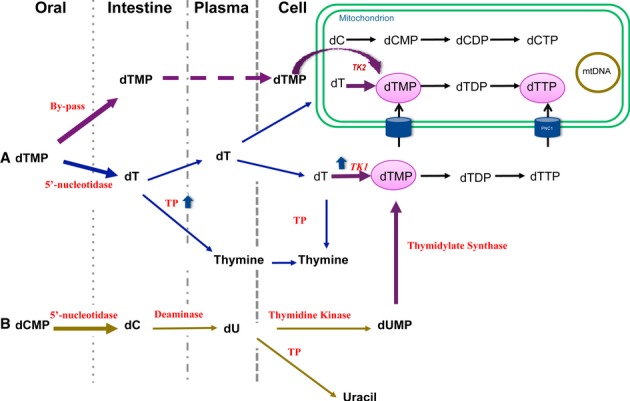
Deoxypyrimidine monophosphates pathways Graphical summary of the pathways modulated by oral gavage dCMP/dTMP treatment. A   dTMP metabolism. dTMP treatment may enter as monophosphate into the mitochondria bypassing the TK2 enzymatic defect as demonstrated by the increased level of dTTP in postnatal day 13 mutant mouse tissues. However, dTMP is also rapidly degraded by 5′-nucleotidase in the small intestine to the nucleoside (dT), which may be processed via three different pathways: (i) phosphorylated by residual Tk2 activity to eventually produce dTTP within mitochondria; (ii) converted to dTMP by cytosolic Tk1; or (iii) catabolized by thymidine phosphorylase (TP). The combination of reduced Tk1 activity in brain and increased thymidine phosphorylase (TP) activity in small intestine after postnatal day 13 may account for the reduced efficacy of the treatment in rescuing the dNTP pool balance after age 13 days. B   dCMP metabolism. dCMP/dTMP treatment did not increase dCTP levels in mitochondria of *Tk2*^*−/−*^ mice suggesting that dCMP administered orally does not enter into mitochondria. Instead, dCMP degraded to nucleoside (dC) may be a source of dTMP as shown in the figure or may be catabolized to uracil by cytosolic TP.

Analyses of plasma and tissue levels of dCMP/dTMP and their metabolites revealed increases in deoxythymidine (dT) and deoxyuridine (dU), but not dCMP or dTMP 30 min after oral gavage treatment. Based on these findings, we hypothesize that dCMP/dTMP can be effective either by rapid and transient bypassing of the enzyme defect and/or by increasing the dT and thymine (T) substrates as documented by measurements of dT/T levels in plasma and tissue and by dNTP pool analysis in 13-day-old mice. In contrast, dCMP is catabolized by deoxycytidine deaminase, the enzyme responsible for dCMP conversion to deoxyuridine monophosphate (dUMP) (Heinemann & Plunkett, 1989; Jansen *et al*, 2011). The dUMP may be converted to dTMP via thymidylate synthase and may contribute to the observed increases in dTTP levels (Fig 7). Because nucleosides are intrinsically unstable, catabolized in vascular and tissue compartments, and ineffectively phosphorylated by mutant TK2, we postulate that treatment with dT and dC may be less effective than dTMP and dCMP in *Tk2* mutant mice.

We previously demonstrated that Tk2 (mitochondrial) activity is constant in the second week of life, whereas cytosolic Tk1 activity decreases significantly between postnatal day 8 and 13 (Dorado *et al*, 2011). The downregulation of Tk1 activity unmasks Tk2 deficiency in *Tk2*^*−/−*^ mice and coincides with the onset of mtDNA depletion causing inexorable organ failure leading to death. In the present work, we demonstrated that oral dCMP and dTMP delayed the reduction in Tk1 activity. Thus, in addition to providing substrates for dNTP synthesis, dCMP/dTMP supplementation in *Tk2*^*−/−*^ mice appears to enhance compensatory Tk1 activity. Nucleotides generated by *de novo* synthesis can enter into mitochondria through carriers that have been previously demonstrated indirectly in the case of dTMP or directly by PNC1 in the case of dTTP (Ferraro *et al*, 2006; Franzolin *et al*, 2012). Once in mitochondria, dTMP and dTTP can be incorporated into replicating mtDNA as demonstrated directly through *in vitro* modulation of Tk1 activity and PNC1 carrier in cells exposed to radiolabeled thymidine (Franzolin *et al*, 2012) and indirectly by our *in vivo* results. The reduced efficacy of dCMP/dTMP after age 29 days may be due to decreased Tk1 activity.

Oral dCMP/dTMP failed to improve dCTP and dTTP levels in brain cytoplasm and mitochondria of 29-day-old mutants that manifested a head tremor, growth plateau, and subsequently died. The head tremor is likely due to cerebellar dysfunction as we noted a severe complex I protein defect in this tissue. The inability of dCMP/dTMP to prevent CNS manifestations is likely attributable to the development of the blood–brain barrier in *Tk2*^*−/−*^ mice after age 13 days. Therefore, intrathecal treatment may be required in the *Tk2*^*−/−*^ mice and *TK2* patients with encephalopathy (Galbiati *et al*, 2006; Gotz *et al*, 2008).

There are several additional factors that contribute to the incomplete efficacy of dCMP/dTMP therapy in the Tk2-deficient mice. First, orally administered dTMP is catabolized by the dramatically increased thymidine phosphorylase activity in the small intestine of 29-day-old mice. Second, supplemental dTMP and dCMP failed to fully correct intra-mitochondrial levels of dTTP and dCTP, indicating that mitochondrial membranes impede dNTP entry; this hypothesis is supported by the low intra-mitochondrial dCTP/dTTP levels with normal cytosolic concentrations of dCTP and dTTP in liver of 29-day-old treated mice. Third, the doses of dTMP/dCMP may have been insufficient, as we have observed dose-related efficacy. Addressing these therapeutic obstacles (e.g. inhibiting TP activity) may lead to enhance effectiveness of dCMP/dTMP treatment in *Tk2*^*−/−*^ mice.

In conclusion, deoxypyrimidine supplementation for Tk2 deficiency is the first effective and safe *in vivo* treatment option that may be readily translated to patient affected by Tk2 mutations. Furthermore, this approach is potentially applicable to individuals with other mitochondrial disorders due to nucleotide pools unbalance.

## Materials and Methods

### Mice

Generation and characterization of *Tk2 H126N* knock-in mice were previously reported (Akman *et al*, 2008). All experiments were performed according to a protocol approved by the Institutional Animal Care and Use Committee of the Columbia University Medical Center and were consistent with the National Institutes of Health Guide for the Care and Use of Laboratory Animals. Mice were housed and bred according to international standard conditions, with a 12-h light, 12-h dark cycle, and sacrificed at 4, 13, and 29 days of age.

Organs (brain, spinal cord, liver, heart, kidney, quadriceps muscle, lung, and gastrointestinal tract) were removed and either frozen in the liquid phase of isopentane, pre-cooled near its freezing point (−160°C) with dry ice or fixed in 10% neutral-buffered formalin and embedded in paraffin using standard procedures. Paraffin-embedded tissue were then stained with hematoxylin and eosin (H&E) for morphological study or processed for immunostaining studies with GFAP, COX I, or complex I subunit as described in detail in the supplemental procedures. All the experiments were performed in at least three mice per group. Both heterozygous and homozygous wild-type mice were considered as control group (*Tk2*^*+*^) as no clinical and biochemical difference were previously described (Akman *et al*, 2008; Dorado *et al*, 2011).

### Treatment administration and experimental plan

Deoxycytidine monophosphate (dCMP) and deoxythymidine monophosphate (dTMP) (Hongene Biotech, Inc.) were administered in 50 μl of Esbilac milk formula for small pets (Pet-Ag) by daily oral gavage to *Tk2* H126N knock-in mice (*Tk2*^*−/−*^) and aged-matched control wild type (*Tk2*^*+*^) using 2 doses, 200 and 400 mg/kg/day, from postnatal day 4 to day 29. At age 29 days, mice were separated from the mother and the treatment was continued by administration of dCMP and dTMP in drinking water using equimolar doses, respectively, of 1.6 and 3.2 mM. A negative control group of untreated *Tk2* mutant and control wild-type mice were weighted and observed closely for comparison.

### Phenotype assessment

To define the degree of safety and efficacy of dTMP/dCMP, we compared survival time, age at onset of disease, type and severity of symptoms, occurrence of side effects, and proportion of treatment termination due to adverse events in treated and untreated *Tk2* mice. General behavior, survival time, and body weights of the mice were assessed daily beginning at postnatal day 4. Videotaping and open-field test with an Opto-Varimetrix-3 sensor system (Columbus Instruments) were performed at 13 and 29 days by counting horizontal and vertical movements, by recording ambulatory and resting time and by measuring the total distance traveled in 10 min.

### Brain histology

Brain and spinal cord samples from 13- to 29-day-old mice were fixed with 10% neutral-buffered formalin and embedded in paraffin using standard procedures. Cerebellum, brainstem, hippocampus, cerebral cortex, and cervical, thoracic and lumbar tracts of the spinal cord were analyzed.

Sections (5 μm thick) were stained with H&E and luxol fast blue to analyze the overall structure of the tissue. Immunostaining with antibodies against GFAP, complex I (NDUFB6), or COX subunit 2 was also performed. Briefly, paraffin-embedded brain and spinal cord slides were deparaffinized, rehydrated, and rinsed in phosphate-buffered saline solution (PBS). To block endogenous peroxidase activity, sections were incubated with 3% hydrogen peroxide in methanol. Slides were then placed in 0.1 M sodium citrate buffer (pH 6.0) and heated in a microwave oven for 15 min, for antigen retrieval. Slides were incubated with mouse anti-GFAP antibody (1:100) (Novocastra. NCL-GFAP-GA5) or mouse monoclonal antibody anti-complex I 17 kDa (NDUFB6) subunit (1:100) (A21359; Molecular Probes) or mouse monoclonal antibody anti-COX subunit 2 (1:100) (clone COX 229, A6404; Molecular Probes) overnight at 4°C. Sections were subsequently rinsed in PBS and incubated with anti-mouse M.O.M. Peroxidase kit, 1:200 dilution for 60 min at room temperature. Immunoreactivity was detected by avidin–biotin complex (ABC) with DAB substrate (Vector Laboratories, Burlingame, CA, USA). Slides were examined by light microscopy using an Olympus BX51 microscope, and images were captured with a QImaging Retiga EXi digital camera, using QCapture software version 2.68.6.

### dNTP pool by polymerase extension essay

Tissues were homogenized on ice in 10 volumes (w/v) of cold MTSE buffer (210 mM mannitol, 70 mM sucrose, 10 mM Tris–HCl pH 7.5, 0.2 mM EGTA, 0.5% BSA) and centrifuged at 1,000 *g* for 5 min at 4°C, followed by three centrifugations at 13,000 *g* for 2 min at 4°C. Supernatant was precipitated with 60% methanol for the mitochondrial fraction and 100% methanol for the cytosolic fraction, kept 2 h at −80°C, boiled 3 min, stored at −80°C (from 1 h to overnight), and centrifuged at 20,800 *g* for 10 min at 4°C. Supernatants were evaporated until dry, and pellet was resuspended in 65 μl of water and stored at −80°C until analyzed. To minimize ribonucleotide interference, total dNTP pools were determined as reported (Ferraro *et al*, 2010; Marti *et al*, 2012a). Briefly, 20 μl volume reactions was generated by mixing 5 μl of sample or standard with 15 μl of reaction buffer [0.025 U/ml ThermoSequenase DNA polymerase (GE Healthcare, Piscataway, NJ, USA) or Taq polymerase (Life Technologies, NY, USA), 0.75 μM 3H-dTTP or 3H-dATP (Moravek Biochemicals), 0.25 μM specific oligonucleotide, 40 mM Tris–HCl, pH 7.5, 10 mM MgCl_2_, 5 mM DTT]. After 60 min at 48°C, 18 ml of reaction were spotted on Whatman DE81 filters, air dried, and washed three times for 10 min with 5% Na_2_HPO_4_, once in distilled water and once in absolute ethanol. The retained radioactivity was determined by scintillation counting.

### Nucleotides measurements by HPLC

Nucleotides concentrations were measured as described (Akman *et al*, 2008) with minor modifications in brain, muscle, and liver. 50 mg of tissue was homogenized in 500 ml of ice-cold 0.5 M perchloric acid and centrifuge at 16,000 × *g* for 10 min at 4°C. The pellets were stored at −80°C for protein measurement, and nucleotides were measured in the resultant supernatant using the Alliance HPLC (Waters Corporation, Milford, MA, USA) with an Alltima C18NUC reverse-phase column (Alltech Associates, Deerfield, IL, USA) and UV detection. After stabilizing the column with the mobile phase, samples (50 ml) were injected onto the HPLC system. The mobile phase consists of 0.2 M ammonium phosphate buffer pH 3.5 (phase A) and 30% methanol in 0.2 M ammonium phosphate buffer, pH 3.5 (phase B). The time schedule for the binary gradient was as previously reported (Ferraro *et al*, 2006). Standard curves for dCTP, dTTP, dTMP, and dCMP were constructed with 15, 30, and 60 mM of each nucleotide. Absorbance of the samples was measured with an UV detector at 260 nm wavelength, and the concentration of each nucleotide in the samples was calculated based on the peak area. Nucleotide levels were expressed in nmol/mg prot.

### Nucleosides measurements by HPLC

Deoxythymidine (dT), deoxyuridine (dU), uracile (U), and thymine (T) levels were assessed by a gradient-elution HPLC method as described previously (Lopez *et al*, 2009; Marti *et al*, 2012b), with minor modifications. Briefly, deproteinized samples were injected into an Alliance HPLC system (Waters Corporation) with an Alltima C18NUC reversed-phase column (Alltech) at a constant flow rate of 1.5 ml/min (except where indicated) using three buffers: eluent A (20 mM potassium phosphate, pH 5.6), eluent B (water), and eluent C (methanol). Samples were eluted over 60 min with a gradient as follows: 0–5 min, 100% eluent A; 5–25 min, 100–71% eluent A, 29% eluent B; 25–26 min, 0–100% eluent C; 26–30 min, 100% eluent C; 30–31 min, 0–100% eluent B; 31–35 min, 100% eluent B (1.5 – 2 ml/min); 35 – 45 min, 100% eluent B (2 ml/min); 45 – 46 min, 100% eluent B (2–1.5 ml/min); 46–47 min, 0–100% eluent C; 47–50 min, 100% eluent C; 50–51 min, 0–100% eluent A; and 51–60 min, 100% eluent A.

Absorbance of the elutes was monitored at 267 nm, and dThd and dUrd peaks were quantified by comparing their peak areas with a calibration curve obtained with aqueous standards. For definitive identification of dT, dU, U, and, T peaks for each sample, we used a second aliquot treated with excess of purified E. coli TP (Sigma) to specifically eliminate dT and dU. The detection limit of this method is 0.05 mmol/l for all nucleosides.

### RT-qPCR: mitochondrial DNA quantification

Real-time PCR was performed with the primers and probes for murine COX I gene (mtDNA) and mouse glyceraldehyde-3-phosphate dehydrogenase (GAPDH, nDNA) (Applied Biosystems, Invitrogen, Foster City, CA, USA) as described using standard curve quantification, in an ABI PRISM 7,000 Sequence Detection System (Applied Biosystems) (Dorado *et al*, 2011). MtDNA values were normalized to nDNA values and expressed as percent relative to wild type (100%).

### Mitochondrial respiratory chain protein levels

Thirty micrograms of whole brain cerebrum or cerebellum extracts was electrophoresed in an SDS-12% PAGE gel, transferred to Immun-Blot™ PVDF membranes (Bio-Rad, Hercules, CA, USA) and probed with MitoProfile® Total OXPHOS Rodent WB Antibody Cocktail of antibodies (MitoSciences, Eugene, OR, USA). Protein–antibody interaction was detected with peroxidase-conjugated mouse anti-mouse IgG antibody (Sigma-Aldrich, St Louis, MO, USA), using Amersham™ ECL Plus western blotting detection system (GE Healthcare Life Sciences, UK). Quantification of proteins was carried out using NIH ImageJ 1.37V software. Average gray value was calculated within selected areas as the sum of the gray values of all the pixels in the selection divided by the number of pixels.

### Mitochondrial respiratory chain enzyme activities by spectrophotometer analysis

Mitochondrial RC enzymes analysis was performed in cerebrum and cerebellum tissues as previously described (DiMauro *et al*, 1987).

### Nucleosides and nucleotides metabolic enzymes

Thymidine phosphorylase and thymidine kinase 1 and 2 activities were measured as previously described (Martí *et al*, 2003; Lopez *et al*, 2009; Dorado *et al*, 2011).

### Statistical methods

Data are expressed as the mean ± SD of at least three experiments per group. Gehan-Breslow-Wilcoxon test was used to compare the survival proportion of each group of mice. Unpaired *t*-test with Welch's correction and Mann–Whitney *U*-test were used to compare 13-day-old *Tk2*^*+*^ versus untreated *Tk2*^*−/−*^, 13-day-old untreated *Tk2*^*−/−*^ versus *Tk2*^*−/−*200dCMP/dTMP^, 29-day-old wild-type versus *Tk2*^*−/−*200dCMP/dTMP^ and *Tk2*^*−/−*400dCMP/dTMP^, for molecular and biochemical studies. Response to treatment was evaluated comparing *Tk2*^*−/−*^ versus *Tk2*^*−/−*200dCMP/dTMP^ at 13 days and *Tk2*^*−/−*200dCMP/dTMP^ versus *Tk2*^*−/−*400dCMP/dTMP^. A *P*-value of < 0.05 was considered to be statistically significant.

**The paper explained**

**Problem**

Mitochondrial DNA depletion syndrome (MDS) is a frequent cause of severe childhood encephalomyopathy characterized molecularly by reduction of mtDNA copy number in tissues and insufficient synthesis of mitochondrial respiratory chain complexes. Mutations in TK2, encoding mitochondrial thymidine kinase 2, frequently cause mtDNA depletion syndrome presenting as fatal myopathy, spinal muscular atrophy (SMA-like) disease, or encephalomyopathy in early childhood. In addition, Tk2 deficiency has been found to cause adult-onset myopathy with progressive external ophthalmoplegia and multiple deletions of mtDNA. We generated and characterized thymidine kinase 2 H126N knock-in mouse model that recapitulates the human infantile encephalomyopathy and further demonstrated that the pathogenesis of the disorder is due to loss of Tk2 enzyme activity and unbalanced deoxynucleotide triphosphate (dNTP) pools with lack of deoxythymidine and deoxycytidine triphosphates.

**Results**

We have demonstrated that ‘molecular bypass therapy’ with orally administered deoxythymidine monophosphate and deoxycytidine monophosphate produces dramatic clinical, molecular, biochemical, and histological improvements in our *Tk2* knock-in mouse model.

**Impact**

Our results reveal, for the first time, *in vivo* efficacy and safety of a molecular therapy that improves downstream metabolic defects in TK2 deficiency. The treatment is potentially translatable to human use; therefore, our work may impact the treatment of patients with this rare devastating disorder. Furthermore, our approach can be extended to other mitochondrial disorders with unbalanced nucleotide pools.

## Supporting information

for this article is available online: http://embomolmed.embopress.org
