# Supplementary material for: Deoxypyrimidine monophosphate bypass therapy for thymidine kinase 2 deficiency
Source: EMBO Mol Med. 2014 Jun 26;6(8):1016–27. doi: 10.15252/emmm.201404092 (PMC4154130; doi:10.15252/emmm.201404092)
Supplement: Supplementary file 1 [file emmm0006-1016-sd1.pdf]

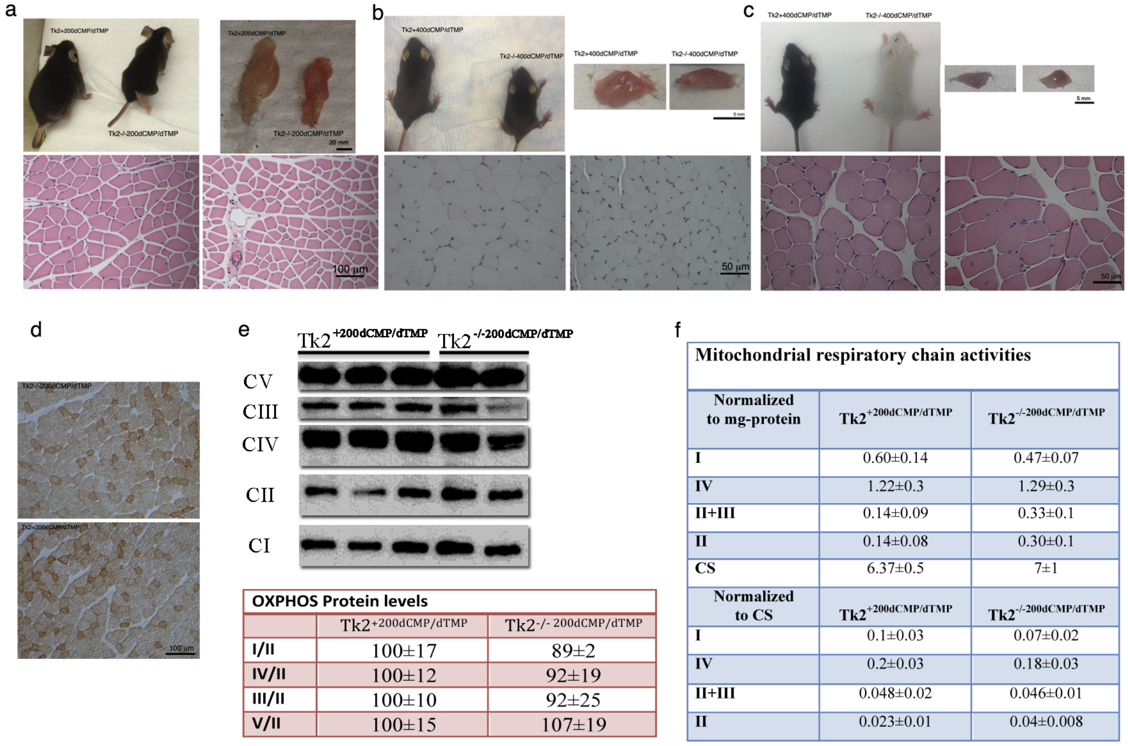

OXPHOS Protein levels

	Tk2+200dCMP/dTMP	Tk2-/- 200dCMP/dTMP
I/II	100±17	89±2
IV/II	100±12	92±19
III/II	100±10	92±25
V/II	100±15	107±19

Mitochondrial respiratory chain activities

Normalized to mg-protein	Tk2+200dCMP/dTMP	Tk2-/-200dCMP/dTMP
I	0.60±0.14	0.47±0.07
IV	1.22±0.3	1.29±0.3
II+III	0.14±0.09	0.33±0.1
II	0.14±0.08	0.30±0.1
CS	6.37±0.5	7±1
**Normalized to CS**	**Tk2+200dCMP/dTMP**	**Tk2-/-200dCMP/dTMP**
I	0.1±0.03	0.07±0.02
IV	0.2±0.03	0.18±0.03
II+III	0.048±0.02	0.046±0.01
II	0.023±0.01	0.04±0.008

Supplementary Figure S1: Neuromuscular phenotype in 29-day old treated mice. a-c) Decreased body weight, quadriceps muscle size, and myofiber diameters in *Tk2*$^{-/-200dCMP/dTMP}$ (right panels) vs *Tk2*$^{+200dCMP/dTMP}$ (a) and in *Tk2*$^{-/-400dCMP/dTMP}$ vs *Tk2*$^{+400dCMP/dTMP}$ (b,c). d) Muscle cytochrome *c* oxidase histochemical activity in *Tk2*$^{-/-200dCMP/dTMP}$ vs *Tk2*$^{+200dCMP/dTMP.}$ e) Representative western blot of mitochondrial proteins in muscle of *Tk2*$^{-/-200dCMP/dTMP}$ vs *Tk2*$^{+200dCMP/dTMP}$ (percents relative to wild-type muscle) (Table). f) Mitochondrial respiratory chain activities *Tk2*$^{-/-200dCMP/dTMP}$ vs *Tk2*$^{+200dCMP/dTMP}$ expressed in micromole/min/mg tissue and normalized to mg-protein and relative to citrate synthase (CS) (mean±SD). No statistically significant differences were detected between *Tk2*$^{-/-200dCMP/dTMP}$ *vs* *Tk2*$^{+200dCMP/dTMP}$ and *Tk2*$^{-/-400dCMP/dTMP}$ *vs* *Tk2*$^{+400dCMP/dTMP}$ samples.

CS= citrate synthase; I=NADH-dehydrogenase; II= succinate dehydrogenase; III= cytochrome *c* reductase; IV= cytochrome *c* oxidase; V= ATP synthase; I+III= NADH cytochrome *c* reductase; II+III= succinate cytochrome *c* reductase.
