# Supplementary material for: Deoxypyrimidine monophosphate bypass therapy for thymidine kinase 2 deficiency
Source: EMBO Mol Med. 2014 Jun 26;6(8):1016–27. doi: 10.15252/emmm.201404092 (PMC4154130; doi:10.15252/emmm.201404092)
Supplement: Supplementary file 2 [file emmm0006-1016-sd2.pdf]

Supplementary Table S1: Long-term treatment in wild-type mice. Body weight (grams) of treated and untreated homozygous (*Tk2*$^{+/+}$) and heterozygous (*Tk2*$^{+/-}$) mice at ages 13, 29, and 60 postnatal days. Statistical analyses were performed with untreated *Tk2*$^{+/+}$ vs milk-fed *Tk2*$^{+/+}$; milk-fed *Tk2*$^{+/+}$ vs *Tk2*$^{+/+200dCMP/dTMP}$; milk-fed *Tk2*$^{+/+}$ vs *Tk2*$^{+/+400dCMP/dTMP}$; untreated *Tk2*$^{+/-}$ vs milk *Tk2*$^{+/-}$; untreated *Tk2*$^{+/-}$ vs *Tk2*$^{+/-200dCMP/dTMP}$; and untreated *Tk2*$^{+/-}$ vs *Tk2*$^{+/-400dCMP/dTMP}$ at P13 and P29. P= postnatal day; Milk= Esbilac milk formula for small pets (Pet-Ag)

	P13	P29	P60
**Untreated *Tk2*$^{+/+}$**	6.6±0.9 (n=10)	17±1.4 (n=2)	23 (n=1)
**Untreated *Tk2*$^{+/-}$**	6.7±1 (n=14)	17.3±2.6 (n=7)	25±5 (n=4)
**Milk *Tk2*$^{+/+}$**	6.6±1.15 (n=3)	16.5±0.7 (n=2)	24 (n=1)
**Milk *Tk2*$^{+/-}$**	6.16±0.5 (n=18)	12.8±1.98 (n=8)	22±2.8 (n=2)
***Tk2*$^{+/+200dCMP/dTMP}$**	7.85±1.14 (n=6)	19.3±1.6 (n=3)	28 (n=1)
***Tk2*$^{+/-200dCMP/dTMP}$**	7.96±1.19 (n=21)	16.5±1.9 (n=12)	21±1 (n=2)
***Tk2*$^{+/+400dCMP/dTMP}$**	7.97±0.3 (n=2)	15.7±3.2 (n=7)	-
***Tk2*$^{+/-400dCMP/dTMP}$**	7.54±1.5 (n=7)	14.9±0.5 (n=2)	24±0 (n=2)
