# Supplementary material for: Deoxypyrimidine monophosphate bypass therapy for thymidine kinase 2 deficiency
Source: EMBO Mol Med. 2014 Jun 26;6(8):1016–27. doi: 10.15252/emmm.201404092 (PMC4154130; doi:10.15252/emmm.201404092)
Supplement: Supplementary file 3 [file emmm0006-1016-sd3.pdf]

Supplementary Table S2– dNTP pools level in tissues. dNTP pools in isolated mitochondria from brain and liver tissues assessed by a polymerase extension assay. Data expressed in pmol normalized to mg-protein (mean±SD). Statistical analyses were performed with untreated *Tk2*$^{-/-}$ *vs* untreated *Tk2*$^{+}$ (P13) and *Tk2*$^{-/-200dCMP/dTMP}$ *vs* *Tk2*$^{+200dCMP/dTMP}$ (P13 and P29). *=p<0.05; **=p<0.005. P= postnatal day

**Brain Mitochondria**	**Untreated *Tk2*$^{+}$ (P13; n=5)**	**Untreated *Tk2*$^{-/-}$ (P13; n=4)**	***Tk2*$^{+200dCMP/dTMP}$ (P13; n=5)**	***Tk2*$^{-/-200dCMP/dTMP}$ (P13; n=5)**	**Untreated *Tk2*$^{+}$ (P29; n=4)**	***Tk2*$^{+200dCMP/dTMP}$ (P29; n=5)**	***Tk2*$^{-/-200dCMP/dTMP}$ (P29; n=8)**
dATP	0.91±0.2	1.51±1	0.85±0.5	1.03±0.8	0.44±0.2	0.86±0.4	0.5±0.3
dTTP	2.52±1	0.67±0.1*	3.55±1	1.52±0.7	1.87±0.9	3.5±1	0.11±0.05**
dGTP	1.06±0.4	1.17±0.7	1.9±1	0.68±0.3	1.3±0.5	1.9±1	0.87±0.6
dCTP	1.99±0.9	3.9±3	3.07±2	1.13±0.5	0.32±0.2	1.2±0.6	0.6±0.2
**Liver Mitochondria**	**Untreated *Tk2*$^{+}$ (P13; n=3)**	**Untreated *Tk2*$^{-/-}$ (P13; n=3)**	***Tk2*$^{+200dCMP/dTMP}$ (P13; n=3)**	***Tk2*$^{-/-200dCMP/dTMP}$ (P13; n=3)**	**Untreated *Tk2*$^{+}$ (P29; n=3)**	***Tk2*$^{+200dCMP/dTMP}$ (P29; n=5)**	***Tk2*$^{-/-200dCMP/dTMP}$ (P29; n=4)**
dATP	1.47±1	1.34±0.8	1.42±0.8	2.6±1	0.32±0.2	0.5±0.3	0.31±0.3
dTTP	0.61±0.08	0.54±0.2	0.67±0.3	0.36±0.3	0.46±0.3	1.2±0.8	0.15±0.04*
dGTP	2.2±2	2.64±1	0.93±0.9	1.2±1	0.58±0.25	0.75±0.4	0.8±0.6
dCTP	2.9±1	1.07±0.8	1.13±0.4	0.56±0.5	0.36±0.3	0.67±0.2	0.04±0.03*
