# Supplementary material for: Deoxypyrimidine monophosphate bypass therapy for thymidine kinase 2 deficiency
Source: EMBO Mol Med. 2014 Jun 26;6(8):1016–27. doi: 10.15252/emmm.201404092 (PMC4154130; doi:10.15252/emmm.201404092)
Supplement: Supplementary file 4 [file emmm0006-1016-sd4.pdf]

**Supplementary Table S3 – Mitochondrial respiratory chain enzyme activities in brain hemisphere.** Mitochondrial respiratory chain enzyme activities in homogenates of brain cerebral hemispheres of 13 and 29 day-old mice. Data expressed in micromole/min/mg tissue and normalized to mg-proteins (mean±SD) or normalized to citrate synthase (CS) activity or to complex II activity (mean±SD). Statistical analyses were performed with untreated $Tk2^{-/-}$ vs untreated $Tk2^{+}$ (P13) and $Tk2^{-/-200dCMP/dTMP}$ vs $Tk2^{+200dCMP/dTMP}$ (P13 and P29). *=p<0.05; **=p<0.005). P= postnatal day

| **Normalized to mg-proteins** | **Untreated *Tk2*$^{+}$ (P13; n=5)** | **Untreated *Tk2*$^{-/-}$ (P13; n=5)** | ***Tk2*$^{+200dCMP/dTMP}$ (P13; n=4)** | ***Tk2*$^{-/-200dCMP/dTMP}$ (P13; n=5)** | ***Tk2*$^{+200dCMP/dTMP}$ (P29; n=4)** | ***Tk2*$^{-/-200dCMP/dTMP}$ (P29; n=6)** |
|---|---|---|---|---|---|---|
| Complex I | 0.05±0.01 | 0.05±0.03 | 0.04±0.0.03 | 0.07±0.03 | 0.24±0.13 | 0.29±0.06 |
| Complex I+III | 0.79±0.1 | 0.88±0.1 | 0.60±0.1 | 0.89±0.4 | 0.62±0.38 | 0.71±0.16 |
| Complex II+III | 0.36±0.04 | 0.38±0.03 | 0.33±0.03 | 0.30±0.06 | 0.47±0.05 | 0.44±0.01 |
| Complex IV | 0.3±0.1* | 0.17±0.05* | 0.17±0.1 | 0.22±0.13 | 1.98±0.29 | 1.55±0.17 |
| Complex II | 0.4±0.1 | 0.51±0.1 | 0.27±0.1 | 0.43±0.06 | 0.98±0.18 | 0.79±0.04 |
| CS | 5.3±1** | 7.5±1** | 6.05±0.8 | 7.2±1 | 7.2±1.2 | 7.09±0.9 |
| **Normalized to CS** | **Untreated *Tk2*$^{+}$ (P13; n=5)** | **Untreated *Tk2*$^{-/-}$ (P13; n=5)** | ***Tk2*$^{+200dCMP/dTMP}$ (P13; n=4)** | ***Tk2*$^{-/-200dCMP/dTMP}$ (P13; n=5)** | ***Tk2*$^{+200dCMP/dTMP}$ (P29; n=4)** | ***Tk2*$^{-/-200dCMP/dTMP}$ (P29; n=6)** |
| Complex I | 0.009±0.003 | 0.007±0.003 | 0.007±0.004 | 0.01±0.005 | 0.03±0.022 | 0.042±0.009 |
| Complex I+III | 0.15±0.05 | 0.11±0.01 | 0.1±0.03 | 0.12±0.056 | 0.08±0.04 | 0.1±0.02 |
| Complex II+III | 0.07±0.014 | 0.05±0.006* | 0.05±0.01 | 0.04±0.01 | 0.066±0.011 | 0.062±0.007 |
| Complex IV | 0.057±0.03 | 0.02±0.008* | 0.028±0.01 | 0.03±0.017 | 0.28±0.06 | 0.22±0.04 |
| Complex II | 0.07±0.02 | 0.069±0.01 | 0.045±0.019 | 0.06±0.017 | 0.14±0.03 | 0.11±0.01 |
| **Normalized to II** | **Untreated *Tk2*$^{+}$ (P13; n=5)** | **Untreated *Tk2*$^{-/-}$ (P13; n=5)** | ***Tk2*$^{+200dCMP/dTMP}$ (P13; n=4)** | ***Tk2*$^{-/-200dCMP/dTMP}$ (P13; n=5)** | ***Tk2*$^{+200dCMP/dTMP}$ (P29; n=4)** | ***Tk2*$^{-/-200dCMP/dTMP}$ (P29; n=6)** |
| Complex I | 0.13±0.05 | 0.11±0.07 | 0.11±0.04 | 0.18±0.1 | 0.25±0.12 | 0.37±0.08 |
| Complex I+III | 2.07±0.6 | 1.75±0.3 | 2.34±0.5 | 2.06±0.9 | 0.74±0.7 | 0.89±0.15 |
| Complex II+III | 0.97±0.25 | 0.76±0.15 | 1.37±0.4 | 0.7±0.05 | 0.50±0.15 | 0.55±0.02 |
| Complex IV | 0.79±0.3 | 0.36±0.1 | 0.66±0.3 | 0.55±0.3 | 2.06±0.4 | 1.96±0.13 |
| CS | 14.2±4.2 | 15.01±3.2 | 19.4±4.6 | 17.05±4.1 | 7.78±3 | 8.9±1.2 |
