# Supplementary material for: Deoxypyrimidine monophosphate bypass therapy for thymidine kinase 2 deficiency
Source: EMBO Mol Med. 2014 Jun 26;6(8):1016–27. doi: 10.15252/emmm.201404092 (PMC4154130; doi:10.15252/emmm.201404092)
Supplement: Supplementary file 5 [file emmm0006-1016-sd5.pdf]

Supplementary Table S4 – Cerebellar mitochondrial respiratory chain enzyme activities.

Mitochondrial respiratory chain enzyme activities in homogenates of whole cerebellum of 13 and 29 day-old mice. Data expressed in micromole/min/mg tissue and normalized to mg-proteins or normalized to citrate synthase (CS) activity or to complex II activity (mean±SD). Statistical analyses were performed with untreated $Tk2^{-/-}$ *vs* untreated $Tk2^{+}$ and $Tk2^{-/-200dCMP/dTMP}$ *vs* $Tk2^{+200dCMP/dTMP}$. *=p<0.05; **=p<0.005) P= postnatal day

Normalized to CS	COX	I	II+III	II	I+III
$Tk2^{+}$	0.24±0.07	0.08±0.03	0.11±0.02	0.11±0.03	0.14±0.03
$Tk2^{-/-}$	0.13±0.07*	0.015±0.01**	0.06±0.02*	0.06±0.01*	0.067±0.05*
$Tk2^{+13\ days\ 200}$	0.24±0.07	0.065±0.04	0.11±0.03	0.11±0.003	0.11±0.06
$Tk2^{-/-13\ days\ 200}$	0.19±0.01	0.036±0.01	0.10±0.02	0.087±0.06	0.11±0.01
$Tk2^{+29\ days}$	0.20±0.02	0.069±0.007	0.072±0.004	0.073±0.008	0.10±0.01
$Tk2^{+\ 29\ days\ 200}$	0.17±0.03	0.049±0.004	0.071±0.02	0.065±0.01	0.11±0.03
$Tk2^{-/-\ 29\ days\ 200}$	0.11±0.03*	0.034±0.01	0.057±0.01	0.080±0.02	0.05±0.005*
$Tk2^{+29\ days}$	0.027±0.002	0.002±0.002	0.039±0.009	0.07±0.01	0.028±0.002
$Tk2^{+29\ days\ 400}$	0.036±0.022	0.005±0.001	0.017±0.012	0.035±0.02	0.027±0.019
$Tk2^{-/-\ 29\ days\ 400}$	0.04±0.024	0.004±0.002	0.02±0.006	0.05±0.01	0.028±0.019
**Normalized to II**	**COX**	**I**	**II+III**	**CS**	**I+III**
$Tk2^{+}$	2.1±0.6	0.74±0.3	1.01±0.1	9.1±2.5	1.31±0.2
$Tk2^{-/-}$	2.1±0.7	0.29±0.2	1.06±0.1	18.5±7.4	0.97±0.6
$Tk2^{+13\ days\ 200}$	2.09±0.2	0.43±0.3	0.98±0.1	9.1±2.5	0.92±0.3
$Tk2^{-/-13\ days\ 200}$	1.7±0.4	0.29±0.1	0.84±0.0.5	8.8±1.8	0.94±0.1
$Tk2^{+29\ days}$	2.8±0.02	0.95±0.003	0.99±0.1	13.7±1.5	1.48±0.08
$Tk2^{+\ 29\ days\ 200}$	2.62±0.38	0.65±0.4	0.97±0.1	15.9±3.6	1.76±0.2
$Tk2^{-/-\ 29\ days\ 200}$	1.43±0.4	0.43±0.2	0.72±0.1	13.3±3.5	0.68±0.2
$Tk2^{+29\ days}$	0.38±0.05	0.031±0.04	0.53±0.04	14±2.2	0.4±0.03
$Tk2^{+29\ days\ 400}$	0.43±0.3	0.14±0.02	0.35±0.03	16±4.3	0.56±0.3
$Tk2^{-/-\ 29\ days\ 400}$	0.47±0.3	0.05±0.06	0.41±0.01	19.4±3.4f	0.5±0.2
