# Supplementary material for: Deoxypyrimidine monophosphate bypass therapy for thymidine kinase 2 deficiency
Source: EMBO Mol Med. 2014 Jun 26;6(8):1016–27. doi: 10.15252/emmm.201404092 (PMC4154130; doi:10.15252/emmm.201404092)
Supplement: Supplementary file 6 [file emmm0006-1016-sd6.pdf]

Supplementary Table S5– Deoxypyrimidine monophosphates metabolism. Plasma level of deoxynucleosides at time 0 and after 30 minutes of oral gavage and thymidine phosphorylase activity in brain, liver and small intestine at 13 and 29 days of age in $Tk2^{+200dCMP/dTMP}$ and $Tk2^{-/-200dCMP/dTMP}$ and aged matched untreated mutant and wild-type. S.I. = small intestine; dUrd= deoxyuridine; Thd= thymidine.

**Plasma Levels of nucleoside metabolites**								
**Mice**	**Age**	**Treatment**	**Dose**	**Time**	**Uracil (µM)**	**Thymine (µM)**	**dUrd (µM)**	**Thd (µM)**
$Tk2^{+}$ (N=1)	13	dCMP+dTMP	80 nmol/kg/day	0	19.1	UND	5.5	1.0
$Tk2^{+}$ (N=3)	13	dCMP+dTMP	80 nmol/kg/day	30	59.3 ±33.9	3.6±3.3	29.0±18.5	71.6±53.3
$Tk2^{-/-}$ (N=1)	13	dCMP+dTMP	80 nmol/kg/day	0	21.2	5.0	4.2	6.8
$Tk2^{-/-}$ (N=3)	13	dCMP+dTMP	80 nmol/kg/day	30	78.0±56.7	5.4±3.5	28.3±9.1	70.4±19.2
$Tk2^{+}$(N=3)	29	dCMP+dTMP	80 nmol/kg/day	0	79.7±20.5	0.1±0.1	2.2±0.1	5.0±5.4
$Tk2^{+}$ (N=2)	29	dCMP+dTMP	80 nmol/kg/day	30	109.3±1.3	68.7±53.3	29.3±13.4	73.4±50.2
$Tk2^{-/-}$ (N=3)	29	dCMP+dTMP	80 nmol/kg/day	30	61.4±10.9	11.2±3.4	18±1.9	30.8±13.2
**Thymidine Phosphorylase activity**								
**Mice**	**Age**	**Treatment**	**Dose**	**TISSUE**	**Activity (nmol/h/mg-proteins)**			
$Tk2^{+}$(N=3)	13	dCMP+dTMP	80 nmol/kg/day	BRAIN	6.2±3.3			
$Tk2^{-/-}$ (N=4)	13	dCMP+dTMP	80 nmol/kg/day	BRAIN	11.6±0.8			
$Tk2^{+}$(N=2)	29	dCMP+dTMP	80 nmol/kg/day	BRAIN	5.9±0.4			
$Tk2^{-/-}$ (N=2)	29	dCMP+dTMP	80 nmol/kg/day	BRAIN	8.0±0.8			
$Tk2^{+}$(N=4)	13	Untreated	-	LIVER	4.9±2.4			
$Tk2^{-/-}$ (N=2)	13	Untreated	-	LIVER	6.1±2.6			
$Tk2^{+}$(N=3)	13	dCMP+dTMP	80 nmol/kg/day	LIVER	9.0±2.9			
$Tk2^{-/-}$ (N=3)	13	dCMP+dTMP	80 nmol/kg/day	LIVER	6.0±3.1			
$Tk2^{+}$(N=6)	29	dCMP+dTMP	80 nmol/kg/day	LIVER	4.9±2.45			
$Tk2^{-/-}$ (N=4)	29	dCMP+dTMP	80 nmol/kg/day	LIVER	4.6±2.5			
$Tk2^{+}$(N=6)	13	Untreated	-	S.I.	26.1±11.0			
$Tk2^{-/-}$ (N=3)	13	Untreated	-	S.I.	28±14			
$Tk2^{+}$(N=2)	13	dCMP+dTMP	80 nmol/kg/day	S.I.	29.6±3.9			
$Tk2^{-/-}$ (N=3)	13	dCMP+dTMP	80 nmol/kg/day	S.I.	30.9±10.6			
$Tk2^{+}$ (N=9)	29	dCMP+dTMP	80 nmol/kg/day	S.I.	278.8±75			
$Tk2^{-/-}$ (N=4)	29	dCMP+dTMP	80 nmol/kg/day	S.I.	244.1±80			
