# Supplementary material for: Deoxypyrimidine monophosphate bypass therapy for thymidine kinase 2 deficiency
Source: EMBO Mol Med. 2014 Jun 26;6(8):1016–27. doi: 10.15252/emmm.201404092 (PMC4154130; doi:10.15252/emmm.201404092)
Supplement: Supplementary file 8 [file emmm0006-1016-sd8.pdf]

Video 1: Effects of dTMP/dCMP in 13-day old mice.
