# Supplementary material for: Deoxypyrimidine monophosphate bypass therapy for thymidine kinase 2 deficiency
Source: EMBO Mol Med. 2014 Jun 26;6(8):1016–27. doi: 10.15252/emmm.201404092 (PMC4154130; doi:10.15252/emmm.201404092)
Supplement: Supplementary file 9 [file emmm0006-1016-sd9.pdf]

Deoxypyrimidine monophosphate bypass therapy for thymidine kinase 2 deficiency

Caterina Garone, Beatriz Garcia-Diaz, Valentina Emmanuele, Luis Carlos Lopez, Saba Tadesse, Hasan O. Akman, Kurenai Tanji, Catarina M. Quinzii and Michio Hirano

Corresponding author: Michio Hirano, Columbia University Medical Center

Review timeline:

Submission date:	20 March 2014
Editorial Decision:	07 April 2014
Revision received:	01 May 2014
Editorial Decision:	07 May 2014
Revision received:	22 May 2014
Accepted:	26 May 2014

Transaction Report:

(Note: With the exception of the correction of typographical or spelling errors that could be a source of ambiguity, letters and reports are not edited. The original formatting of letters and referee reports may not be reflected in this compilation.)

Editor: Roberto Buccione

1st Editorial Decision 07 April 2014

Thank you for the submission of your manuscript to EMBO Molecular Medicine. We have now received comments from the three Reviewers whom we asked to evaluate your manuscript

You will see that all three Reviewers are quite supportive of you work, although they do raise a few issues that prevent us from considering publication at this time.

I will not dwell into much detail, as the evaluations are self-explanatory. I would like, however, to mention that the main points are essentially requests for clarification on presentation, results and experimental details. One leitmotif among the Reviewers is clearly on the doses and length of treatment and consequent potential transferability to humans.

In conclusion, while publication of the paper cannot be considered at this stage, we would be pleased to consider a suitably revised submission, provided that the Reviewers' concerns are fully addressed.

Please note that it is EMBO Molecular Medicine policy to allow a single round of revision only and that, therefore, acceptance or rejection of the manuscript will depend on the completeness of your responses included in the next, final version of the manuscript.

As you know, EMBO Molecular Medicine has a "scooping protection" policy, whereby similar findings that are published by others during review or revision are not a criterion for rejection. Although I clearly do not foresee such an instance in this case, I do ask you to get in touch with us after three months if you have not completed your revision, to update us on the status. Please also contact us as soon as possible if similar work is published elsewhere.

I look forward to seeing a revised form of your manuscript as soon as possible.

***** Reviewer's comments *****

Referee #1 (Comments on Novelty/Model System):

The model organism recapitulates very well the biochemical impairment as well as the clinical outcomes of the human Tk2 deficiency syndrome. The novelty and medical impact are high because it is the first nucleotide supplementation for mtDNA depletion syndromes to show beneficial effects in vivo. The technical quality is adequate. Improving data presentation and better explanation of statistical comparisons would be helpful.

Referee #1 (Remarks):

The manuscript by Garone and colleagues reports the beneficial effects of dCTP/dTTP supplementation therapy in a knock in mouse with defective Tk2. The mouse presents severe encephalomyopathy due to lack of mitochondrial nucleotides, resulting in respiratory chain defects. Untreated Tk2-/- mice died by two weeks of age, but the nucleotides replacement afforded a dose dependent delay of disease onset and extension of lifespan, up to 34 days, with the highest dose of 400 mg/Kg/day by gavage.
This is the first in vivo evidence that nucleotide supplementation can ameliorate the symptoms as well as the biochemical defects of Tk2 deficiency, and more in general of any of the known forms of nuclear encoded mtDNA depletion syndromes. Thus, the findings have significant implications for molecular therapy of mitochondrial disorders caused by nucleotide imbalance.
While the results are very encouraging, as the authors correctly note, there are still barriers that need to be overcome. Importantly, the rescue of mitochondrial nucleotide levels was incomplete and tissue specific. In particular, the CNS, which is severely affected in the disease, did not show an increase of mitochondrial at 29 days of age, likely because the maturation of the BBB prevented the passage of exogenous nucleotides from blood. Therefore, the encephalopathy worsened rapidly after this age, leading to fatal outcome. Intrathecal administration will be tested in the future to bypass the BBB.
The results presented convincingly demonstrate that the supplementation therapy has a beneficial effect at multiple levels, from nucleotide pools in plasma and tissue early on to improved phenotype. The interpretation of the biochemistry based on the nucleotide utilization pathways in figure 5 is logical.
The main problem with the manuscript, in my view, is that it is sometimes difficult to follow the flow, mostly due to the organization of the data, in figures and tables. It would be helpful to reorganize the findings in a different way, whereby the reader is not forced to go back and forth to compare figures and tables and especially data presented in different figures in a sparse order. It may be advisable to focus the description of the results on the most relevant findings, such as the change in nucleotide levels in blood and tissues, mtDNA levels, and the respiratory chain function, in addition to the phenotypic findings. Nevertheless, it is noteworthy that the authors have thoroughly described all the measurements performed, including the negative data, therefore providing a very complete picture of the treatment outcomes.

Specific points:

1) In the tables and legends, it is generally unclear what is compared and why. The legend should be made clearer.
2) The safety of the treatment is assesses in a very short study in wild type mice (only 30 days). If the treatment were to be used chronically in humans, a longer safety trial would be needed.
3) The n of animals in the study in figure 1 (weight and survival) and the table don't seem to match. The n needs to be clarified better.
4) Many comparisons are between wild type (Tk2+) and Tk2-/- and others between treated and untreated Tk2-/-, at different ages, different tissues, etc. It is really hard to follow. There must be a way to make the result section flow better, especially inregards to histology and histoenzymatic findings.

5) The scheme in figure 5 suggests that dTTP is rescued through at least two pathways, but it does not explain how dCTP would be improved.

Referee #2 (Comments on Novelty/Model System):

• Easy to read.
• Well executed study.
• Potential therapeutic application.
• Good that the mouse model presents with the same phenotype as humans, which is not always the case. Despite this, it does not guarantee that the same response to the treatment will happen in humans.

Referee #2 (Remarks):

Garone et al - Deoxypyrimidine monophosphate bypass therapy for thymidine kinase 2 deficiency

This work is from a well established and well respected group. It is a concise paper with a very specific point to make that is delivered clearly in this manuscript.

The fact that the knock-in and knock-out mutant mice show a very similar phenotypic presentation as is seen in humans is interesting and potentially present more useful models than many mice attempting to mimic mito-disease. This increases the possibility that their observations could be used to develop a therapeutic intervention, although the efficacy in humans may be very different as may be the effects on life expectancy, however, this does not detract from an interesting approach to resolve the biochemical defect. Please see comment below on dosage.
It is becoming clear that the nucleotide balance rather than just total amounts is an important sensor for mitochondrial dysfunction, with profound consequences.

• This is a thorough and well implemented investigation with a very clear hypothesis and well described goals.
• The text is written in clear, nicely phrased language.
• The data are well described, honestly evaluated and well presented in clear figures of high enough resolution to be able to see relevant details.

Minor points
• I don't know whether the doses used on mice are realistically convertible for humans or if these amounts may cause a deleterious effect in humans that might not be anticipatable form mouse metabolism. A more detailed comment on this might be a useful inclusion.
• The 'THE PAPER EXPANDED' should I believe read 'the paper explained'. I think this may have been the only typographical error.
• I am not entirely sure if the legend format conforms to the journal style but this is an editorial point rather than one reflecting scientific content.

Referee #3 (Comments on Novelty/Model System):

Technical quality and novelty are high because a novel line of treatment is demonstrated in vivo. The medical impact is medium because TK2 deficiency is a rare -orphan disease.

Referee #3 (Remarks):

The authors investigate deoxyribonucleoside monophosphate treatment of TK2 in a knock-in mouse model of the mitochondrial DNA (mtDNA) depletion syndrome disorder-TK2 deficiency. They show that dTMP and dCMP supplementation ameliorate the symptoms and increase mtDNA content

and mitochondrial respiratory chain activates demonstrating a possible pharmacological treatment for this devastating disorder.
This study is interesting, thorough and extensive. I have only a few comments and questions.
General comments
1-As the pyrimidine monophosphates are negatively charged, is there evidence (from literature) that they penetrate the cell membranes as such vs the possibility that the nucleotides are de-phosphorylated to deoxynucleosides and subsequently re-phosphorylated in the cytoplasm? i.e could deoxyribonucleosides be effective as well?
2- Could long term treatment induce deoxyribonucleotide imbalance in the nuclear/cytoplasmic compartment i.e causing genomic instability. Was there any evidence of malignancies, and/or chromosomal aberrations in the treated animals?
3- 0.2-0.4g/kg per day is seems like a very high dose were lower doses investigated? If yes were they effective?
Results section
1- Table E1 spell out weight grams?? What is P? days? What is "milk" what kind of milk?
2- Result section - table E2 P13 was the brain cCTP higher in the -/- mouse and decreased with treatment?? There are also other inconsistencies in the table how can these be explained?.
3-table E2 The values in the cytosol seem to be near detection limit (due to diluted sample?) and this part is therefore questionable. Could it be removed/explained?.
4- table E3 E4 what is "tissue" whole tissue homogenate or isolated mitochondria? Maybe normalization to II (nuclear encoded) would give a clearer picture?
5-ALL graphs - define y axis (some are defined some not) and explain the meaning of the graphs in the figure legends (some are explained some are not)
6- fig 2 and 3 seem to be somewhat redundant could they be combined?
6- fig 4 and in the text explain "TP" also explain the rationale why it was measured
Methods section
1 - In the section "dNTP pool by polymerase extension essay" was the dry pellet resuspended in 200 ml?

1st Revision - authors' response 01 May 2014

<u>Reviewer 1:</u> We appreciate this reviewer's insightful comments (highlighted in italics).
"This is the first in vivo evidence that nucleotide supplementation can ameliorate the symptoms as well as the biochemical defects of Tk2 deficiency, and more in general of any of the known forms of nuclear encoded mtDNA depletion syndromes. Thus, the findings have significant implications for molecular therapy of mitochondrial disorders caused by nucleotide imbalance". "*The main problem with the manuscript, in my view, is that it is sometimes difficult to follow the flow, mostly due to the organization of the data, in figures and tables. It would be helpful to reorganize the findings in a different way, whereby the reader is not forced to go back and forth to compare figures and tables and especially data presented in different figures in a sparse order".*

We agree that parts of the text and figures are difficult to follow. As suggested, we have modified figure order and organization of the data. We have improved the manuscript by addressing the reviewer's specific points as described below.

Specific points:
1) In the tables and legends, it is generally unclear what is compared and why. The legend should be made clearer.
We thank the reviewer for noting the lack of information about statistical analysis. We generally compared aged-matched untreated vs treated mutants, untreated wild-type vs. treated mutant mice, and treated wild-type vs. treated mutants. Because untreated mutant mice died at age 13-21 days-old, we could not compare treated vs. untreated 29 day-old mutants. We list specific group comparisons in each legend.

2) The safety of the treatment is assesses in a very short study in wild type mice (only 30 days). If the treatment were to be used chronically in humans, a longer safety trial would be needed.

We agree that for chronic human use, a longer safety trial would be useful. We have extended studies in a small number of wild-type mice to age 60 days as shown in Table E1, but even longer term studies are likely to be required and are beyond the scope of this manuscript.

3) The n of animals in the study in figure 1 (weight and survival) and the table don't seem to match. The n needs to be clarified better.

We thank the astute reviewer for noting the discrepancy. The animals in the Figure 1 (weight and survival) are included in the Table E1. The n do not match because only littermates of Tk2 mutants were used for the survival curve while weight data include a second additional group of wild-type mice.

4) Many comparisons are between wild type (Tk2+) and Tk2-/- and others between treated and untreated Tk2-/-, at different ages, different tissues, etc. It is really hard to follow. There must be a way to make the result section flow better, especially in regards to histology and histoenzymatic findings.

We agree with the reviewer; therefore, we specify comparison used for statistical analysis in each figure legend. Furthermore, to improve the flow of result section, we have modified the organization of the figures .

5) "The scheme in figure 5 suggests that dTTP is rescued through at least two pathways, but it does not explain how dCTP would be improved".

We thank the reviewer for pointing out the inadequate explanation of the pathways presented in figure 5 (Figure 7 in the current version). We have expanded the figure legend to indicate more clearly that both dCMP and dTMP treatments are source of dTTP while neither is an effective source of dCTP. According with this hypothesis, we also modified the related statement in the abstract.

Reviewer 2:

Minor points
1) *I don't know whether the doses used on mice are realistically convertible for humans or if these amounts may cause a deleterious effect in humans that might not be anticipatable form mouse metabolism. A more detailed comment on this might be a useful inclusion.*

We thank the reviewer for this interesting comment about the translation to human use. As mentioned in the Discussion of our manuscript, the highest dose tested in an in vitro study is 1200µM (Bulst S et al., 2009), which corresponds to 150 mg/kg/day if dCMP and dTMP are completely absorbed through the gastrointestinal tract. Bulst and colleagues did not observe negative effects. We did not test lower doses but rather started with a higher dose (200 mg/kg/day) because we anticipated that incomplete absorption and catabolism of dCMP and dTMP would severely limit their bioavailability in vivo. In fact, our data demonstrate that high-dose dCMP/dTMP produces relatively small effects on mitochondrial dNTP pools; therefore, we do not think testing lower doses be informative. We did not observe any side effects in our mouse treated with 200 mg/kg/day; therefore, we expect this dose will be safe for human use. We have noted in the manuscript the rationale for testing this dose in the Tk2 knockin mice.

We have already obtained FDA emergency IND approvals for compassionate use in two of our patients with Tk2 deficiency as well as ethical committee approvals for use in two patients in Europe. We started the treatment with an initial dose of 100mg/kg/day and, after 3 month of observation, we have increased the dose to 200 mg/kg/day without any overt side-effects.

2) "*The 'THE PAPER EXPANDED' should I believe read 'the paper explained'. I think this may have been the only typographical error".*
We apologize for the typographical error, which we have corrected.

3) "*I am not entirely sure if the legend format conforms to the journal style but this is an editorial*

point rather than one reflecting scientific content".
We have strived to follow the format for figure legends as described in the Author Guidelines for EMBO Molecular Medicine. We await editorial feedback regarding this point.

Reviewer 3:

General comments
1) *As the pyrimidine monophosphates are negatively charged, is there evidence (from literature) that they penetrate the cell membranes as such vs the possibility that the nucleotides are de-phosphorylated to deoxynucleosides and subsequently re-phosphorylated in the cytoplasm? i.e could deoxyribonucleosides be effective as well?*

We thank the reviewer for the interesting comment. Our results provide indirect evidence that dTMP and dTTP can reach mitochondria. At the same time, the reviewer is correct that prior to entering cells, nucleotides may be de-phosphorylated to nucleosides and after entry into cells re-phosphoryated. In fact, we postulate that thymidine may be entering cells and phosphorylated by Tk1, thus nucleosides may be contributing to the efficacy of dCMP/dTMP therapy. Cytosolic Tk1 in replicating cells converts thymidine into monophosphate while additional cytosolic kinases convert dTMP to dTTP. Both dTMP and dTTP can enter mitochondria via two previously reported carriers (Ferraro P et al., 2006; Franzolin E et al., 2012). Nevetheless, Tk1 activity is reduced in post-mitotic cells and as a consequence, less dTMP and dTTP can reach the mitochondria thereby accounting for the diminished efficacy of dCMP/dTMP in Tk2 deficient mice after post-natal day 29. Furthermore, rapid catabolism of nucleosides (e.g. thymidine catabolism by thymidine phosphorylase) may also limit this potential therapy. Despite these potential limitations, we are currently assessing nucleoside therapy in our Tk2 knockin mice.

2) Could long term treatment induce deoxyribonucleotide imbalance in the nuclear/cytoplasmic compartment i.e. causing genomic instability. Was there any evidence of malignancies, and/or chromosomal aberrations in the treated animals?

We did not see any evidence of malignancies in our studies. We did not screen for chromosomal aberrations in the treated animals; however, CGH-array in dGK deficient cell line treated with dAMP and dGMP showed no chromosomal defects.

3) 0.2-0.4g/kg per day is seems like a very high dose were lower doses investigated? If yes were they effective?

We agree with reviewers 1 and 3 who noted the lack of rationale for the chosen doses. Please see our response to reviewer 1 regarding this issue.

Results section
1) Table E1 spell out weight grams?? What is P? days? What is "milk" what kind of milk?

We thank the reviewer for noting the missing information. We specify weight, P, and type of milk in the legend.

2) Result section - table E2 P13 was the brain cCTP higher in the -/- mouse and decreased with treatment?? There are also other inconsistencies in the table how can these be explained?

As shown in Figure 7 (previously Figure 5) and explained in details in the revised figure legend, both dCMP and dTMP are sources of dTTP. We identified an increased level of dTTP at postnatal day 13 while the relative amount of dCTP appeared equal or reduced in treated mice.

3) table E2 The values in the cytosol seem to be near detection limit (due to diluted sample?) and this part is therefore questionable. Could it be removed/explained?
We agree that those values may be questionable due to diluted samples and normalization to proteins measured in buffer that containing 0.5% of BSA. Therefore, we decide to remove the data obtained with cytosol.

4) table E3 E4 what is "tissue" whole tissue homogenate or isolated mitochondria? Maybe normalization to II (nuclear encoded) would give a clearer picture?

We used whole tissue homogenate. As recommended, we have added data normalized to Complex II; however, the additional analyses did not significantly alter the results.

5) ALL graphs - define y axis (some are defined some not) and explain the meaning of the graphs in the figure legends (some are explained some are not)

We thank the reviewer for noting the missing information and we agree that a better explanation of the graphs would help to follow their meaning. Therefore, the legends were expanded to better clarify the figure.

6) fig 2 and 3 seem to be somewhat redundant could they be combined?

We modified the figure organization combining them by study performed (histology, biochemistry, molecular analyses) in order to improve the flow of the paper.

7) fig 4 and in the text explain "TP" also explain the rationale why it was measured

We have added the previously missing rationale for measuring thymidine phosphorylase in the paper.

Methods section

In the section "dNTP pool by polymerase extension essay" was the dry pellet resuspended in 200 ml?

We thank the reviewer for noting the error. We now describe the correct volume of fluid used to resuspend the dry pellet generated in the polymerase extension assay.

References:

Bulst S, Abicht A, Holinski-Feder E, Muller-Ziermann S, Koehler U, Thirion C, Walter MC, Stewart JD, Chinnery PF, Lochmuller H, Horvath R (2009) In vitro supplementation with dAMP/dGMP leads to partial restoration of mtDNA levels in mitochondrial depletion syndromes. Hum Mol Genet 18:1590-1599.

Ferraro P, Nicolosi L, Bernardi P, Reichard P, Bianchi V (2006) Mitochondrial deoxynucleotide pool sizes in mouse liver and evidence for a transport mechanism for thymidine monophosphate. Proc Natl Acad Sci U S A 103:18586-18591.

Franzolin E, Miazzi C, Frangini M, Palumbo E, Rampazzo C, Bianchi V (2012) The pyrimidine nucleotide carrier PNC1 and mitochondrial trafficking of thymidine phosphates in cultured human cells. Exp Cell Res 318:2226-2236.

2nd Editorial Decision 07 May 2014

Thank you for the submission of your revised manuscript to EMBO Molecular Medicine. We have now received the enclosed reports from the referees that were asked to re-assess it. The reviewers are now fully supportive and I am pleased to inform you that we will be able to accept your manuscript pending final editorial amendments:

Please submit your revised manuscript within two weeks.

I look forward to reading the revised version of your manuscript as soon as possible.
