# Supplementary material for: Deoxypyrimidine monophosphate bypass therapy for thymidine kinase 2 deficiency
Source: EMBO Mol Med. 2014 Jun 26;6(8):1016–27. doi: 10.15252/emmm.201404092 (PMC4154130; doi:10.15252/emmm.201404092)

Figure 4 Panel D: Brain 13 days untreated *Tk2*$^{+}$ (W), untreated *Tk2*$^{-/-}$ (M), *Tk2*$^{+200dCMP/dTMP}$ (WT), *Tk2*$^{-/-200dCMP/dTMP}$(MT)

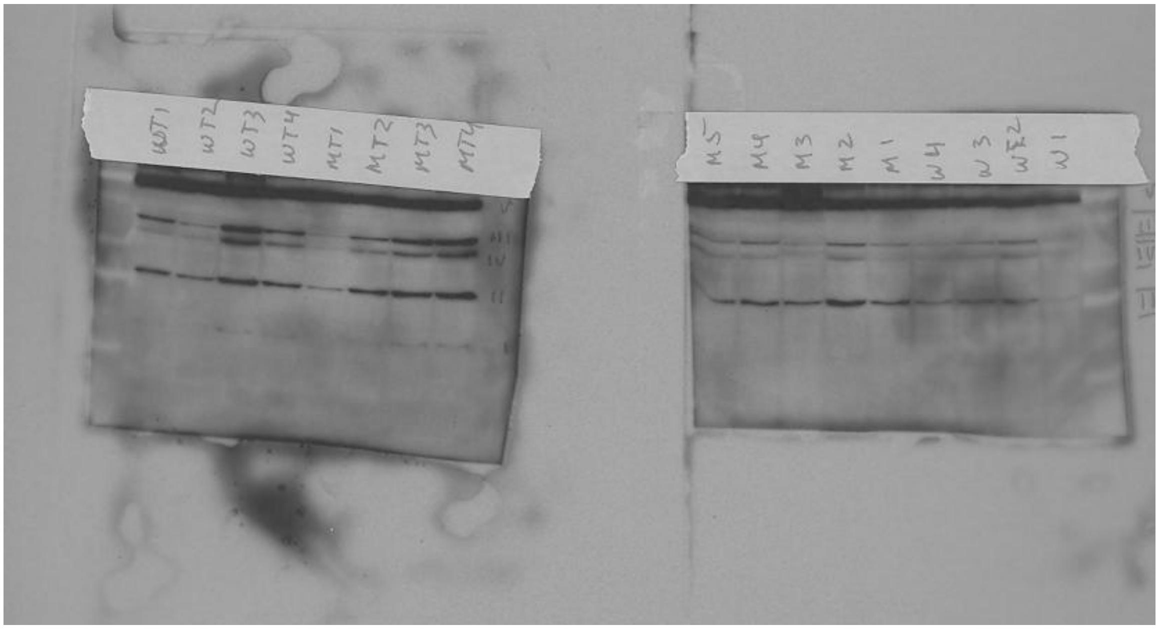

Figure 4 Panel D Brain 29 days

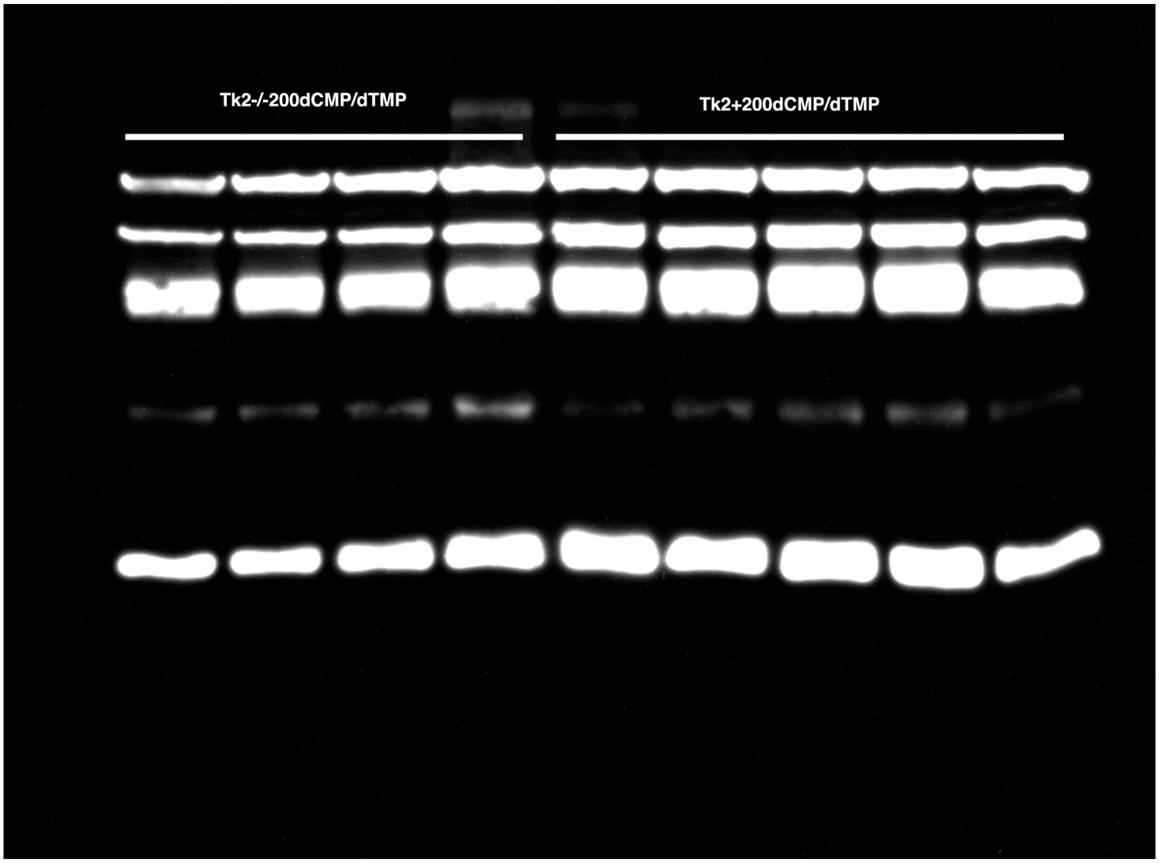

Figure 4 Panel E Cerebellum 13 days

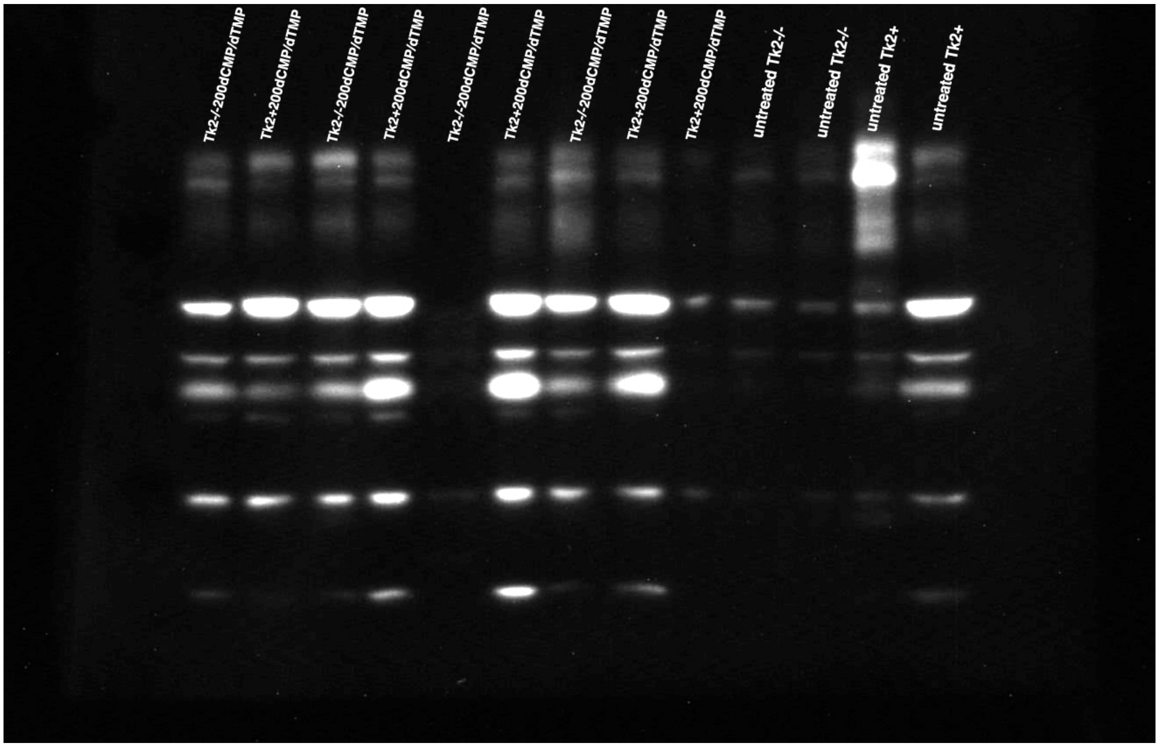

Figure 4 Panel E Cerebellum 29 days

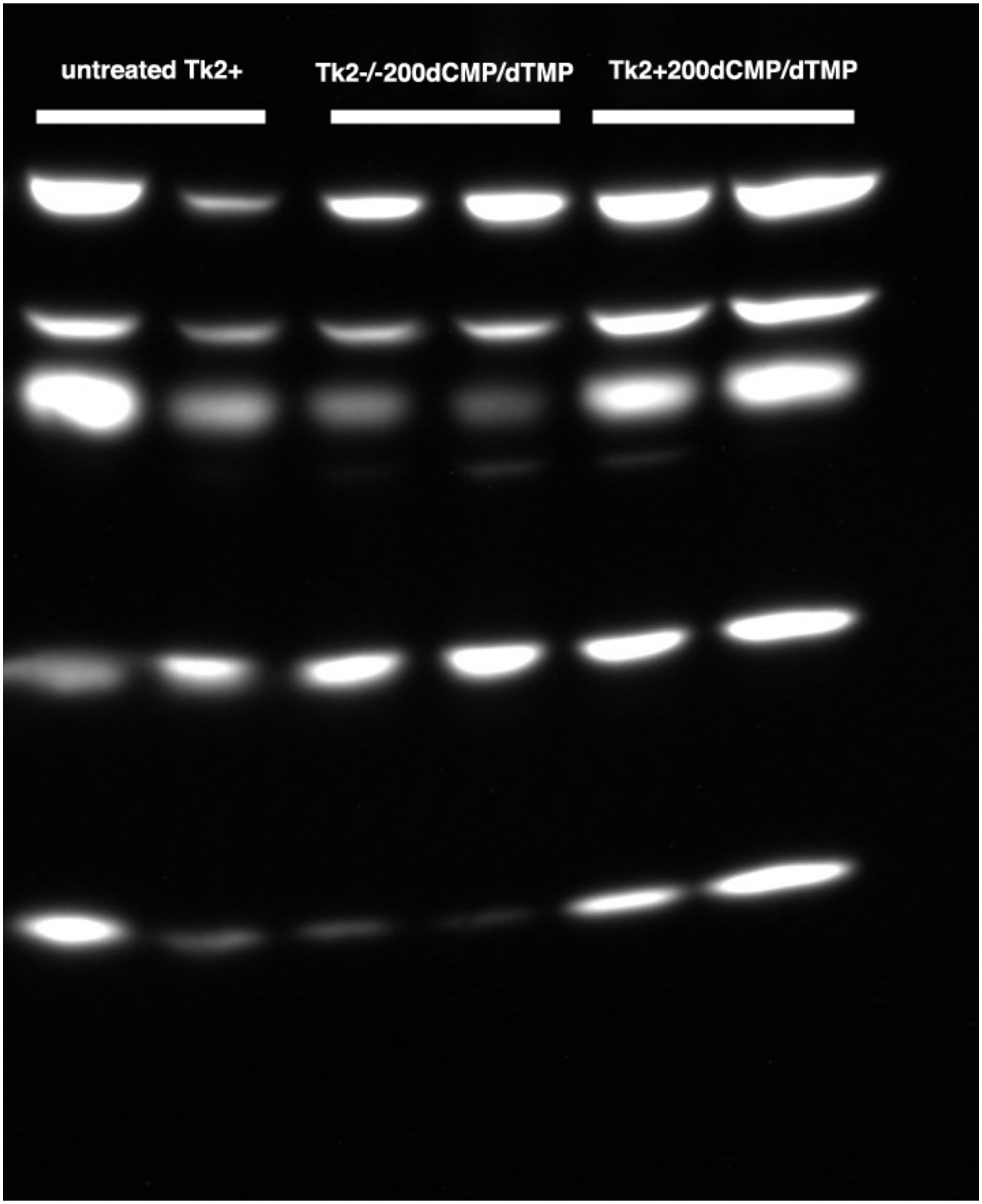

Figure 4 Panel E Cerebellum 29 days

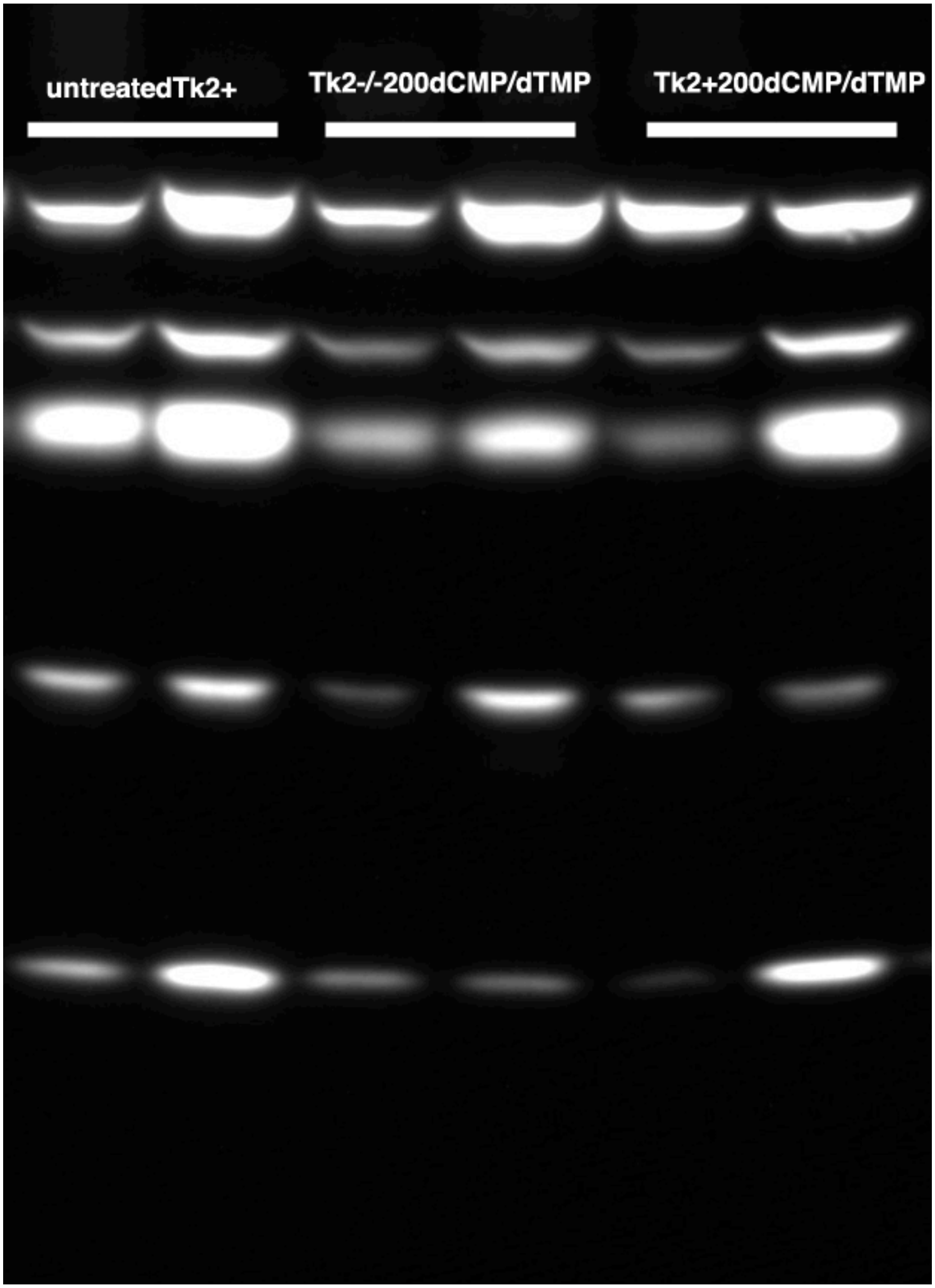

Figure 4 Panel E Cerebellum 29 days

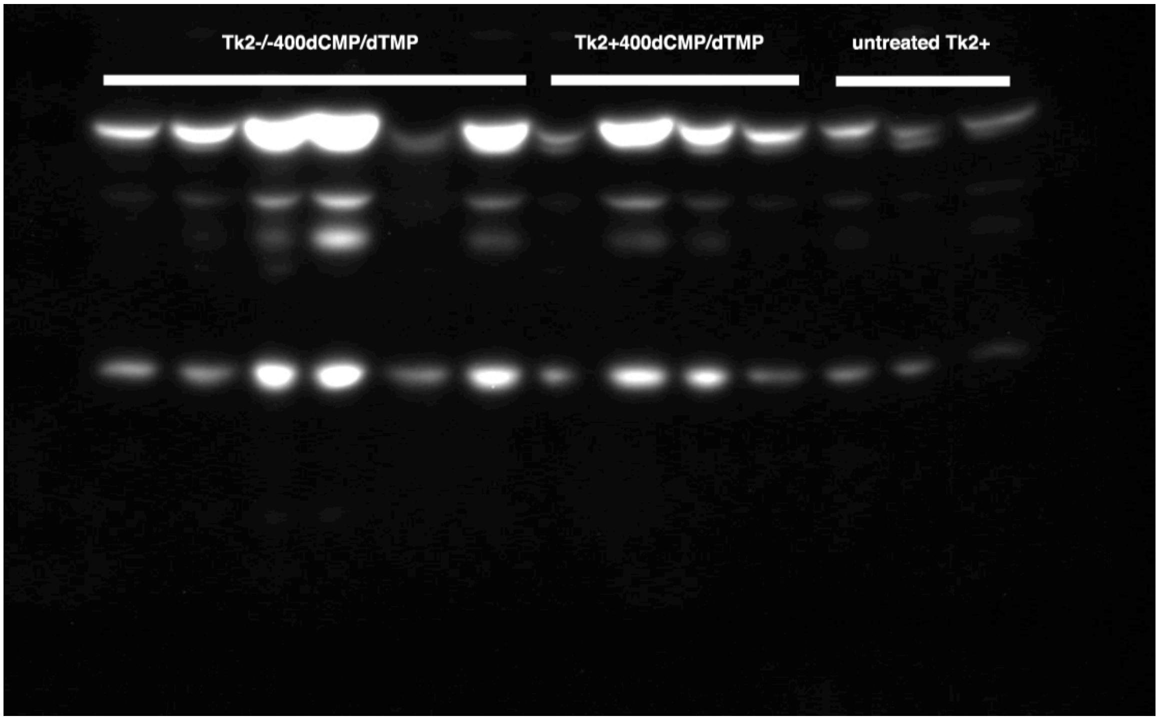

Supplement: Supplementary file 10 [file emmm0006-1016-sd10.pdf]
